# Lithiation‐Driven LiCrSe_2_ Shell Growth on Metallic CrSe_2_ Core Governs the Plateau–Slope Behavior

**DOI:** 10.1002/advs.202523702

**Published:** 2026-02-27

**Authors:** Weihao Li, Johannes Döhn, Xiao Han, Licheng Zhang, Dave M. Pickup, Xiong Xiao, Yidong Miao, Arun Kumar Samuel, Emily R. Draper, Alan V. Chadwick, Stephen Sproules, Stephen Cottrell, Alan J. Drew, Changhua An, Axel Groß, Alexey Y. Ganin

**Affiliations:** ^1^ School of Chemistry University of Glasgow Glasgow UK; ^2^ Institute of Theoretical Chemistry Ulm University Ulm Germany; ^3^ Petrochemical Research Institute PetroChina Beijing P. R. China; ^4^ School of Physical and Chemical Sciences Queen Mary University of London London UK; ^5^ School of Physical Sciences University of Kent Canterbury UK; ^6^ Tianjin Key Laboratory of Organic Solar Cells and Photochemical Conversion School of Chemistry and Chemical Engineering Tianjin University of Technology Tianjin P. R. China; ^7^ School of Materials and Chemical Engineering Xuzhou University of Technology Xuzhou P. R. China; ^8^ ISIS Pulsed Neutron and Muon Source Rutherford Appleton Laboratory Didcot UK; ^9^ Helmholtz Institute Ulm (HIU) for Electrochemical Energy Storage Ulm Germany

**Keywords:** CrSe_2_, core‐shell lithiation, lithium‐ion batteries, layered transition metal dichalcogenides

## Abstract

The growing demand for high‐performance lithium‐ion batteries necessitates the development of cathode materials that combine high capacity, structural stability, and rapid charge–discharge capability. First‐principles calculations predict that layered CrSe_2_ possesses a robust framework capable of accommodating one Li^+^ per formula unit while intrinsically supporting fast Li‐ion diffusion. Muon spin rotation (µ^+^SR) measurements validate this prediction, revealing fast Li^+^ diffusion in pre‐lithiated CrSe_2_. Consistent with these findings, electrochemical testing demonstrates a reversible capacity of 125.3 mAh g^−1^ at 0.1 C, approaching the theoretical value of 127.7 mAh g^−1^, with stable cycling and good rate capability. In operando X‐ray diffraction and electrochemical impedance spectroscopy further reveal a reversible topotactic transition and a lithiation‐driven core‐shell evolution during cycling. These results show that lithiation‐induced conductivity changes govern the electrochemical behavior of CrSe_2_, highlighting its potential as a high‐performance cathode for LIBs. This study provides new insight into intercalation processes in layered transition‐metal chalcogenides and informs the design of fast‐charging electrodes.

## Graphical abstract

Layered CrSe_2_ is investigated as a lithium‐ion battery cathode combining fast Li^+^ diffusion and structural reversibility. Calculations and experiments reveal a single topotactic intercalation process with a characteristic plateau–slope profile governed by lithiation‐induced conductivity changes. A core‐shell lithiation evolution underpins its high‐rate capability and cycling stability.

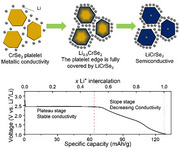

## 1 Introduction

Fast charging remains a major challenge for lithium‐ion batteries (LIBs) and a key barrier to widespread adoption of electric vehicles [1, 2, 3, 4]. While battery capacity and energy density have improved significantly [5, 6, 7, 8], slow charging rates still limit user acceptance and practical deployment. Achieving fast‐charging capability requires not only optimized electrolytes [5, 9, 10, 11] and advanced manufacturing strategies [1, 12, 13] but, more critically, electrode materials that can intrinsically support rapid and reversible Li^+^ insertion without structural degradation or polarization losses. To meet these requirements, electrode hosts must combine high Li^+^ diffusivity, sufficient electronic conductivity, and robust frameworks capable of tolerating repeated ion migration.

Layered transition‐metal dichalcogenides (TMDs) have emerged as promising candidates for high‐rate electrode materials owing to their open van der Waals gaps, strong in‐plane covalency, and intrinsic metallic conductivity [14, 15, 16, 17]. In particular, Ti‐ [18, 19], V [16, 20, 21], and Zr‐based [22, 23] TMDs are well known for their ability to undergo topotactic Li^+^ (de)intercalation while maintaining structural integrity during cycling. In contrast, chromium‐based chalcogenides have received comparatively little attention despite their rich electronic structures and higher Cr^3+^/Cr^4+^ redox potentials [24, 25, 26, 27], which could enable both strong electronic coupling and reversible redox activity. The mixed‐anion compound CrSeS has recently been shown to accommodate one Li^+^ per formula unit and deliver exceptionally high charge–discharge rate performance in LIBs [28]. This outstanding kinetic behavior has been attributed to the expanded interlayer spacing and enhanced polarizability introduced by selenium, which together lower the Li^+^ migration barrier and facilitate ultrafast ion transport [28]. These results suggest that selenium incorporation plays a decisive role in accelerating Li^+^ diffusion within layered Cr‐based hosts, suggesting that fully selenide analogues could exhibit even faster Li^+^ dynamics and superior rate performance.

Building on this insight, it is logical to examine binary CrSe_2_, which contains only selenium and therefore maximizes anion size and polarizability effects [29, 30, 31, 32]. The larger and more deformable Se^2−^ anions can effectively screen electrostatic interactions, facilitating Li^+^ migration through the layered framework and potentially enhancing rate capability beyond that of CrSeS. Using the predictions of a recently suggested descriptor for ion mobility [33, 34] this high mobility is also consistent with the relatively low electronegativity of Se compared to, e.g., oxygen. Previous µ^+^SR studies on pre‐lithiated CrSe_2_ have already revealed fast Li^+^ diffusion kinetics at room temperature [35], underscoring its potential as a high‐rate host. Furthermore, CrSe_2_ has also previously demonstrated promising performance in potassium‐ion batteries by us, achieving its theoretical capacity at 0.1 C and retaining 85% capacity at 1 C [36], highlighting its structural robustness and favorable diffusion kinetics across multiple alkali systems. However, despite decades of research on intercalation chemistry in layered transition‐metal dichalcogenides, the electrochemical lithiation behavior of CrSe_2_ itself has never been systematically investigated. Its electronic and structural response to Li insertion, and the extent to which Se polarizability influences conductivity and diffusion, remain completely unknown. Uncovering these mechanisms is essential for understanding Cr‐based TMDs and for guiding the design of next‐generation fast‐charging electrode materials that combine rapid ion transport with robust structural reversibility.

In this work, we investigate the lithiation dynamics of layered CrSe_2_ by integrating periodic density functional theory (DFT) calculations within in operando and ex situ experiments. DFT predicts that Li^+^ occupies octahedral sites within a stable *P*‐3*m*1 lattice, driving a metallic‐to‐semiconducting transition that terminates at LiCrSe_2_. These predictions are experimentally verified through in operando X‐ray diffraction, X‐ray absorption spectroscopy, electron microscopy, and µ^+^SR analysis. Electrochemical measurements reveal a distinct plateau–slope profile within 1.0–3.0 V, governed by conductivity evolution rather than multiple phase transitions. Combined structural and impedance analyses identify a conductivity‐driven core‐shell evolution, in which a metallic CrSe_2_ core becomes progressively encapsulated by a semiconducting LiCrSe_2_ shell during lithiation. These findings demonstrate that the plateau–slope behavior of CrSe_2_ originates from dynamic electronic reconfiguration, establishing it as a representative model for conductivity‐controlled intercalation in layered chalcogenides.

## 2 Results and Discussion

### 2.1 DFT Predictions of Thermodynamic Lithiation

**FIGURE 1:**
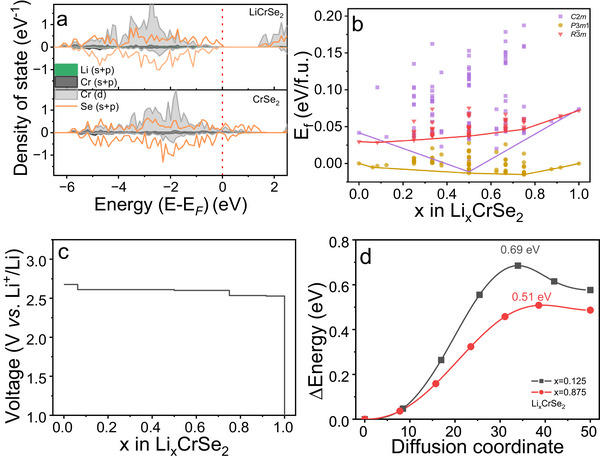
(a) Electronic density of states of LiCrSe_2_ and CrSe_2_. (b) Calculated formation energies of Li_x_CrSe_2_ for different site occupations as explained in more detail in the Supporting Information. (c) DFT‐predicted intercalation voltage profile of CrSe_2_ during lithiation. (d) Li‐ion energy barriers of Li_x_CrSe_2_ for low (x = 0.125) and high (x = 0.875) lithium‐vacancy phases corresponding to the diffusion path from octahedral to tetrahedral site.

Comprehensive DFT investigations were performed on the entire Li_x_CrSe_2_ system including calculations on the electronic structure, structural stability and barriers for Li diffusion. DFT has been shown to provide reliable results for properties of batteries [37]. A thorough discussion on the underlying methodology including a structural benchmark for six different DFT flavors can be found in the Supporting Information and Table S1. Since both CrSe_2_ and LiCrSe_2_ can be synthesized as layered compounds, the corresponding phases were taken as starting point for the computational investigations. The calculated electronic density of states (Figure 1a) shows that pristine CrSe_2_ is metallic, with the Fermi level intersecting strongly hybridized Cr 3d and Se 4p bands consistent with previous reports [36, 38]. Upon lithiation to LiCrSe_2_, electrons donated by Li occupy antibonding Cr‐Se orbitals, which lower the density of states near the Fermi level, open a small band gap, and transform the material from metallic to semiconducting. The substantial orbital overlap implies possible participation of both Cr and Se in charge compensation during lithiation.

Once the delithiated and lithiated phases were established, convex‐hull calculations were conducted to evaluate the thermodynamic stability of possible intermediate compositions in the Li_x_CrSe_2_ system (Figure 1b) Additional to the *P*‐3*m*1 phase for LiCrSe_2_, the *R*‐3*m* and *C*2/*m* structures, known to be stable for NaCrSe_2_ [39] and KCrSe_2_ [40], were examined as possible Li‐intercalation frameworks. Several intermediate configurations for 0.0 ≤ x < 0.08 and for 0.5 < x ≤ 1.0 are predicted to be stable or within the stability range (given the methodological inaccuracy) all of which consistently retain *P*‐3*m*1 symmetry. The lattice spacing jumps from *c* = 5.9 Å at x = 0.08 to *c* = 6.3 Å at x = 0.5 where it remains roughly constant until full lithiation indicating only a single significant structural transition at x = 0.5 for the system. The phase stability of the configurations within 0.5 < x ≤ 1.0 is likely to be driven by charge ordering effects as this region is characterized by only subtle structural changes. No additional low‐energy configurations were identified outside the *P*‐3*m*1 framework, confirming that the layered lattice remains topotactically stable during Li insertion.

Within the *P*‐3*m*1 framework, the preferred Li‐occupation site was further examined to clarify the intercalation mechanism. High‐symmetry octahedral and tetrahedral sites between Se layers were considered, as prior studies indicate that their energy difference can be small [41]. The DFT results (Figure S1) show that octahedral coordination is consistently more stable than tetrahedral occupation across all Li contents. Configurations with Li in tetrahedral sites lie significantly above the convex‐hull line, particularly at higher Li contents, indicating that tetrahedral occupation is energetically unfavorable. Although Li_x_CrSe_2_ (x > 1) has not been previously reported, analogous layered systems such as VS_2_ and TiS_2_ can accommodate a second Li ion once the octahedral sites are fully occupied [42]. However, for CrSe_2_ DFT calculations (Figure S2) reveal that lithiation beyond LiCrSe_2_ does not yield a stable Li_x_CrSe_2_ (x > 1) phase. Instead, additional Li insertion destabilizes the structure and drives decomposition into Li_2_Se and a Cr‐rich residue. These findings indicate that Li intercalation in CrSe_2_ is confined to octahedral sites and terminates at LiCrSe_2_, beyond which further Li uptake triggers a conversion‐type reaction.

Based on the predicted stable phases of Li_x_CrSe_2_ (Figure 1b), the calculated voltage profile exhibits a nearly constant plateau, centered around 2.6 V versus Li^+^/Li (Figure 1c). Although several intermediate phases were predicted from the convex‐hull analysis, their voltage differences are very small, resulting in an almost flat voltage curve in which multiple transitions are not distinguishable in battery testing.

To further examine the kinetic aspect of Li transport, nudged elastic‐band (NEB) calculations were performed at low (x = 0.125) and high (x = 0.875) Li contents, representing dilute and nearly filled states of Li_x_CrSe_2_. The calculated diffusion barriers are 0.69 eV at x = 0.125 and 0.51 eV at x = 0.875, indicating that Li migration becomes slightly easier at higher Li concentration. These barriers are only moderately higher than those reported for Li_x_CrSSe (Figure S3), suggesting that although Li diffusion in CrSe_2_ is somewhat slower, the kinetics remain comparable. Given that CrSSe has been reported to exhibit exceptional rate performance [28], CrSe_2_ is expected to display similarly favorable kinetics, motivating its synthesis and electrochemical characterization to verify the predicted lithiation behavior. Although a lower barrier was initially anticipated due to the larger and more polarizable Se^2−^ anions, the slightly higher value obtained here suggests that stronger Cr‐Se covalency counterbalances this effect, leading to comparable rather than enhanced Li^+^ mobility. Furthermore, the electronegativity of Se (2.60) and S (2.69) is very similar which according to a recently suggested descriptor for ion mobility in crystalline materials based on the so‐called migration number [33, 34] should also yield very similar migration barriers.

### 2.2 Structural, Electronic, and Morphological Characterization CrSe_2_ and LiCrSe_2_

**FIGURE 2:**
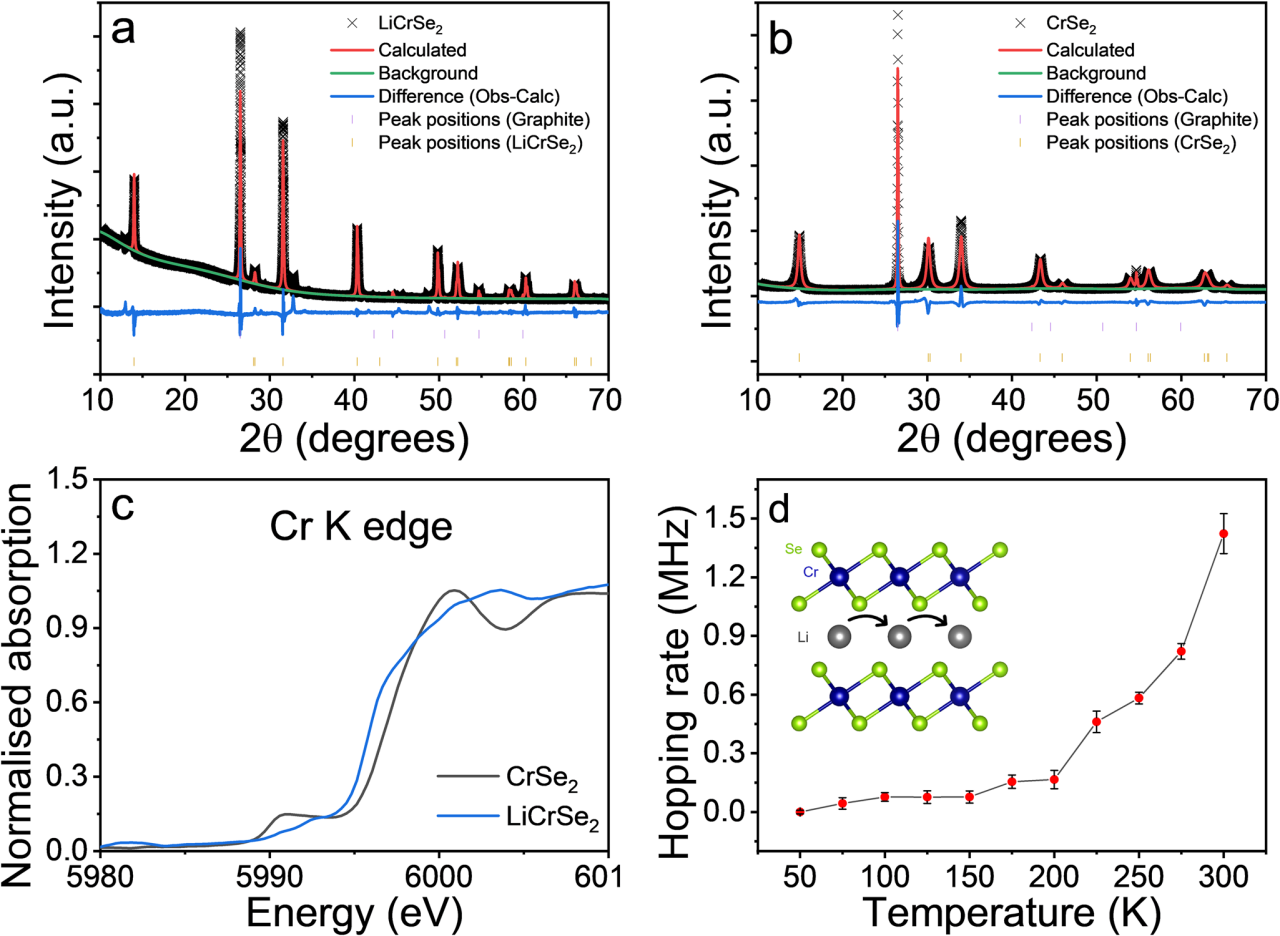
Le Bail refinements of experimental PXRD patterns (Cu Kα) for (a) LiCrSe_2_ and (b) CrSe_2_. Measured data are shown as black crosses, calculated profiles as red lines, and different curves as blue lines. Magenta vertical bars indicate the expected Bragg reflection positions of the phase, (c) Cr K‐edge XANES profile of CrSe_2_ and LiCrSe_2_, and (d) Temperature dependence of the Li‐ion hopping rate (ν_Li_) from µ^+^SR measurements.

To experimentally validate the above DFT predictions, CrSe_2_ was synthesized and comprehensively characterized before electrochemical testing. CrSe_2_ mixed with 10 wt.% graphite (hereafter referred to as CrSe_2_) was synthesized, following our previous reports [36, 38]. The resulting powder product is air‐stable and was used instead of LiCrSe_2_ to ensure compatibility with standard electrode fabrication procedures. Powder X‐ray Diffraction (PXRD) data (Figure 2a,b) collected on CrSe_2_ and LiCrSe_2_ match the reported crystal structure (space group *P*‐3*m*1), as confirmed by the agreement between experimental and modelled profiles. The refined lattice parameters of CrSe_2_: *a* = 3.3956(6) Å and *c* = 5.9168(10) Å, and LiCrSe_2_: *a* = 3.65127(5) Å and *c* = 6.29616(10) Å agree well with literature values (Tables S2 and S4), verifying that both phases crystallize in the expected structure without impurity peaks.

Having confirmed the structural integrity of both compounds, their local electronic structures and oxidation state evolution were probed using X‐ray absorption near‐edge structure (XANES) spectroscopy (Figure 2c; Figure S4). Both CrSe_2_ and LiCrSe_2_ adopt edge‐sharing [CrSe_6_] octahedra, but the Cr─Se bond length expands from 2.47 Å in CrSe_2_ to 2.54 Å in LiCrSe_2_ (Table S4), consistent with Cr reduction upon lithiation. The Cr K‐edge of CrSe_2_ appears at higher energy than that of LiCrSe_2_, consistent with a higher Cr valence in the CrSe_2_ phase. However, both edge positions lie below those of Cr^4+^ and Cr^3+^ reference oxides, evidencing strong Cr─Se covalency arising from hybridized Cr 3d‐Se 4p states [43, 44]. At the Se K edge, CrSe_2_ exhibits a pre‐edge feature at 12660.2 eV and a sharp absorption edge at 12669.4 eV. Upon lithiation, the pre‐edge intensity decreases, and the edge shifts to 12667.4 eV, reflecting partial Se reduction. These spectral changes corroborate the DOS (Figure 1a), confirming strong Cr‐Se hybridization and a transition from metallic CrSe_2_ to semiconducting LiCrSe_2_. The combined Cr‐ and Se‐edge shifts indicate the participation of both elements in the (de)lithiation process.

The microstructure and morphology of CrSe_2_ were investigated using scanning electron microscopy (SEM) and high‐resolution transmission electron microscopy (HRTEM). SEM images (Figure S5) reveal the characteristic stacked‐platelet morphology typical of layered transition‐metal dichalcogenides. Energy‐dispersive X‐ray (EDX) mapping confirms uniform Cr and Se distributions across large areas without elemental segregation, yielding a Cr:Se ratio of 1:2.02 ± 0.02. HRTEM images (Figure S6) show thin, plate‐like crystallites (∼500 nm in lateral dimension) with well‐defined lattice fringes near the platelet edges, confirming high crystallinity. Selected‐area electron diffraction (SAED) patterns obtained along the [001] zone axis display sharp reflections for the (100) and (001) planes, corresponding to d‐spacings of 3.3957 and 5.9182 Å, respectively, in excellent agreement with PXRD results. Fast Fourier transforms (FFT) of HRTEM images further confirm the [001] lattice orientation, while slight in‐plane variations suggest weak stacking disorder between layers. Local EDX mapping of individual platelets reveals strong spatial overlap between Cr and Se signals, with a Se:Cr ratio of 2.01 ± 0.01, consistent with bulk measurements.

While XANES and structural analyses verify the hybrid redox behavior and crystallographic stability predicted by DFT, they do not directly probe Li^+^ mobility, which critically governs the kinetic performance of CrSe_2_ electrodes. To evaluate intrinsic Li^+^ diffusion independent of electrode architecture or electrolyte effects, muon‐spin‐relaxation (µ^+^SR) spectroscopy was employed. This technique quantifies Li^+^ hopping rates in pre‐intercalated compounds [35, 45, 46]. µ^+^SR measurements were performed on as‐synthesized LiCrSe_2_ using the EMU instrument at the ISIS Neutron and Muon Source (more details in Supporting Information and Figure S7). The temperature‐dependent hopping rate increases steadily up to 300 K (Figure 2d), with the onset of mobility between 150 and 175 K, similar to previous reports [35]. The jump frequency was calculated with an Arrhenius behaviour and an activation energy of 74(8) meV (Figure S8). At room temperature, the hopping rate exceeds those of benchmark layered oxides such as Li_x_CoO_2_ [47], LiFePO_4_ [48], and LiNi_0.33_Co_0.33_Mn_0.33_O_2_ [49], confirming that LiCrSe_2_ possesses intrinsically fast Li^+^ diffusion kinetics. Given this superior intrinsic ion‐transport behavior, LiCrSe_2_ is a promising candidate for further electrochemical testing in practical battery configurations.

### 2.3 Electrochemical Behavior of CrSe_2_

**FIGURE 3:**
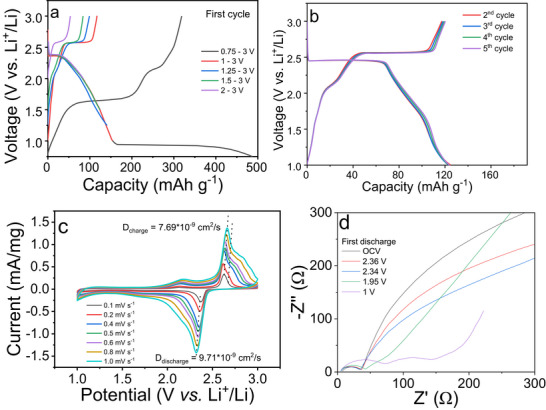
(a) GCD profiles for the 1st cycle of CrSe_2_ recorded at different lower cut off voltages (0.75, 1, 1.25, 1.5, and 2 V), (b) GCD curves (from 2nd cycle) of CrSe_2_ within the voltage window of 1–3 V at 0.1C, (c) CV curves of CrSe_2_ at various scan rates (0.1–1 mV s^−1^) in the voltage range of 1–3 V, with calculated diffusion coefficients for charge and discharge processes. And (d) Electrochemical impedance spectrum measured at different potentials.

To evaluate the Li‐storage behavior of CrSe_2_ and determine an appropriate operating voltage, Swagelok‐type half‐cells were assembled using Li metal as the counter electrode and 1 M LiPF_6_ in EC/DMC (1:1 v/v) as the electrolyte. The galvanostatic charge–discharge (GCD) curves in Figure 3a show how the initial capacity change depending on the potential cut off during the first cycle. However, below about 0.9 V the material undergoes a conversion reaction, which leads to a sharp increase in capacity and a distinctive voltage plateau. The overall discharge capacity reaches 482 mAh g^−1^, far exceeding the theoretical value of 127.7 mAh g^−1^ corresponding to one Li^+^ per CrSe_2_. Such an unusually high capacity cannot arise from further Li intercalation, since DFT predicts that lithiation beyond LiCrSe_2_ is thermodynamically unstable and leads to decomposition into L_2_Se and a Cr‐rich residue (Figure S2). The low‐voltage plateau is therefore attributed to a Li─Se conversion reaction, analogous to that observed in CrS_2_ and CrSSe below ∼1.0 V [28, 42]. The inset photograph in Figure S9 shows the reddish discoloration of the separator after discharge, indicative of soluble Se‐containing conversion products. Consistent with this irreversible process, the first discharge delivers 482 mAh g^−1^ whereas the first charge capacity is limited to 318 mAh g^−1^. The discharge capacity decays to 447 mAh g^−1^ at the second cycles (Figure S9).

To confirm that the sloping region observed above 1.0 V originates from intercalation rather than a conversion process, additional GCD measurements were performed with various higher cutoff voltages of 1.0, 1.25, 1.5, and 2.0 V. As shown in Figure 3b and Figures S10 and S11, the discharge–charge profiles obtained within these voltage windows are nearly identical, and more than 99% of the capacity was retained over the first five cycles. The discharge capacity increases as the cutoff is lowered from 2.0 to 1.0 V, from 64.87 to 125.3 mAh g^−1^. Within the 1.0–3.0 V range, CrSe_2_ delivers 125.3 mAh g^−1^, very close to the theoretical value of 127.7 mAh g^−1^ corresponding to one Li^+^ per CrSe_2_. These results demonstrate that the plateau at ∼2.6 V originates from a fully reversible intercalation process rather than from any conversion‐type reaction. Cycling tests at 0.5 C (Figure S10) further show that discharging to 1.0 and 1.25 V retains approximately 85% of the initial capacity after 50 cycles, whereas cells with higher cutoffs maintain about 94%. Balancing capacity and reversibility, the 1.0–3.0 V window was therefore adopted for all subsequent electrochemical and structural analyses of the plateau–slope behavior, as it enables full theoretical capacity while preventing the irreversible Li─Se conversion reaction observed below 1.0 V.

Starting from the 2nd cycle within the optimized voltage range of 1.0–3.0 V, the discharge curve of CrSe_2_ shows two distinct regions, where a plateau at around 2.6 V and a gradual slope extending to 1.0 V contribute equally to the total capacity (Figure 3b). The plateau region is in excellent agreement with the DFT results (Figure 1c) whereas the predicted voltages for 0.5 < x ≤ 1.0 contrast the experimental findings. We speculate that the applied meta‐GGA functional does not describe the effects of the Cr‐Se hybridization very accurately. The corresponding cyclic voltammetry (CV) curves (Figure 3c; Figure S12) display only one pair of well‐defined redox peaks at 2.36 V (cathodic) and 2.62 V (anodic), representing a single, reversible intercalation‐deintercalation process between CrSe_2_ and LiCrSe_2_. No additional peaks are observed within the 1.0–3.0 V window The peaks remain sharp and nearly symmetric even at higher scan rates, shifting slightly to 2.32 and 2.67 V at 1.0 mV s^−1^ due to mild polarization. The linear relationship between peak current (I_p_) and the square root of the scan rate (*v* ^0.5^) (Figure S13) confirms that Li^+^ transport is diffusion controlled. The calculated diffusion coefficients are 9.71 × 10^−9^ cm^2^ s^−1^ for lithiation and 7.69 × 10^−9^ cm^2^ s^−1^ for delithiation, where CrSe_2_ exhibits slightly larger diffusion coefficients than CrSSe of 4.56 × 10^−9^ cm^2^ s^−1^ and 3.24 × 10^−9^ cm^2^ s^−1^ [28]. Despite this difference, the experimental diffusion coefficients of CrSe_2_ is comparable to other transition metal dichalcogenides (Table S5). Rate performance tests (Figure S14) further validate the favorable Li^+^ kinetics in CrSe_2_, which retaining 63% of its capacity at 1 C relative to 0.1 C. Compared with CrSSe, which maintains about 50% of its capacity even at 200 C [28], CrSe_2_ exhibits slower rate performance, consistent with its slightly higher Li^+^ migration barrier and lower diffusion coefficients.

Electrochemical impedance spectroscopy (EIS) was carried out during the first discharge to monitor the evolution of charge‐transfer resistance (Figure 3d; Figure S15). At open‐circuit voltage, the Nyquist plot exhibits a large semicircle that significantly contracts as the potential enters the plateau region (2.36–2.34 V), indicating stable and relatively low interfacial resistance. The low‐frequency response remains nearly unchanged, suggesting that Li^+^ diffusion is not rate‐limiting in this regime. As lithiation proceeds into the sloping region (1.95–1.0 V), the high‐frequency semicircle gradually enlarges, revealing a progressive increase in charge‐transfer resistance and a decline in electronic conductivity. At deep discharge (1.0 V), the resistance reaches its maximum and an additional low‐frequency feature emerges, evidencing sluggish interfacial kinetics in fully lithiated LiCrSe_2_ [50]. In contrast, the DFT‐calculated density of states for Li_x_CrSe_2_ (Figure S16) shows that the system remains metallic up to x = 0.92. However, upon lithiation the number of states at E_F_ decreases significantly (especially above x = 0.75), leaving only few accessible electronic states in the conduction band which may imply reduce electronic conductivity, until the band gap fully opens at x = 1.0. In addition, temperature‐dependent resistivity measurements were performed on pressed pellets of CrSe_2_ and LiCrSe_2_. Pristine CrSe_2_ exhibits negligibly low resistance, while LiCrSe_2_ shows a temperature‐dependent decrease in resistivity (Figure S17), consistent with semiconducting behavior as predicted by DFT calculations. In conclusion, the reduction in conductivity already begins within the sloping stage, rather than only at complete lithiation as could have been predicted by theory. The discrepancy between the experimental GCD profile and the DFT‐predicted voltage plateau suggests that the transition in CrSe_2_ occurs earlier, around x ≈ 0.5 rather than x = 0.92 as predicted, prompting further investigation through in operando and spectroscopic analyses discussed in Section 2.4.

### 2.4 Structural Evolution of CrSe_2_ During Electrochemical Testing

**FIGURE 4:**
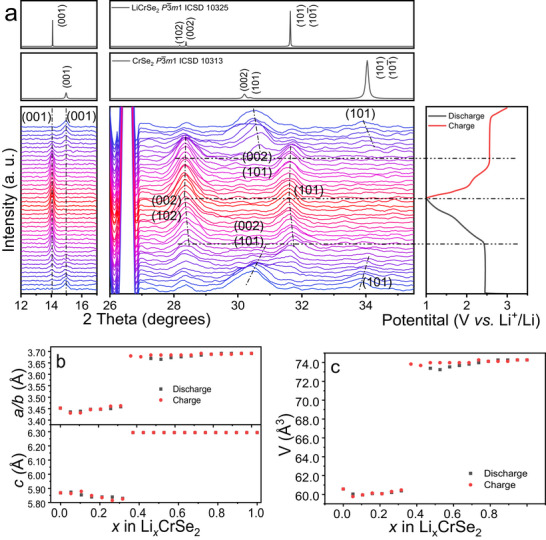
(a) Diffraction patterns from the *in operando* XRD experiment. The right panel shows cell voltage as a function of time at second cycle, which is aligned with the diffraction patterns. Every other pattern was shown here while the intensity was sequentially offset. The cells were charged and discharged between 1.0 and 3.0 V at a rate of C/0.1 for 1 cycle. The simulated CrSe_2_ and LiCrSe_2_ patterns are on the top of in‐operand XRD. (b) Evolution of lattice parameters *a*/*b* and *c* calculated from the *in operando* XRD during the first charge/discharge process, and (c) Unit cell volume calculated from the lattice parameters *a*/*b* and *c*.

The structural evolution of CrSe_2_ during lithiation was monitored using an *in operando* XRD setup similar to that previously employed for CrSe_2_ in potassium‐ion battery studies [36]. Measurements were carried out over the first two discharge–charge cycles, and the resulting contour maps are shown in Figure 4a and Figure S18. The Bragg reflections observed at 14.9° and 34.0° were assigned to the (001) and (101) planes of CrSe_2_, respectively. The (002) and (102) reflections overlap at approximately 30.07°, serving as reference peaks. These features agree well with the simulated XRD pattern of standard CrSe_2_ (Figure 4a; Figure S19). Upon lithiation, a complete disappearance of the original CrSe_2_ reflections and the emergence of new diffraction peaks of LiCrSe_2_ particularly at x ≈ 0.5 in Li_x_CrSe_2_ indicate a transition mechanism. The newly formed peaks at 14.1°, 28.4°, and 31.6° were assigned to the (001), mixed (002)/(102), and (101) planes of the LiCrSe_2_ phase, matching the simulated pattern in Figure 4a and Figure S20. During the recharge process, the peak evolution occurred in the opposite direction to the discharge one, representing a reversible transition between CrSe_2_ and LiCrSe_2_ within 1.0–3.0 V.

Benefiting from the high symmetry of the *P*‐3*m*1 space group in both CrSe_2_ and LiCrSe_2_, structural changes during cycling can be effectively extracted through the evolution of key diffraction reflections. Specifically, the position of the (101) peak is determined by both *a*‐ and *c*‐axis lattice parameters, while the *c*‐axis can be independently monitored through the position of the (001) reflection (details in Supporting Information, Formulas 1 and 2). From the fully delithiated CrSe_2_ of the second cycle (3.0 V), the extracted lattice parameters are *a* = 3.452 Å and *c* = 5.869 Å, while the fully lithiated CrSe_2_ at the end of the second discharge (1.0 V) shows expanded values of *a* = 3.691 Å and *c* = 6.294 Å. These values agree well with both the chemically synthesized CrSe_2_ and LiCrSe_2_ in this work and previously reported data (Tables S2 and S3). The evolution of the lattice parameters during cycling, extracted from Figure 4a, is summarized in Figure 4b,c. The *a*‐axis remains nearly constant at ∼3.452 Å during the plateau stage of discharge (x < 0.5), then expands gradually and stabilizes at 3.691 Å upon further lithiation. During charging, it fully returns to its original value. In contrast, the c‐axis remains unchanged at ∼5.869 Å during the early discharge, then increases to 6.294 Å at deeper lithiation and stays constant thereafter as has been predicted by DFT. Compared with other *P*‐3*m*1 materials, the *c*‐axis expansion in CrSe_2_ exhibits 7.67%, which is slightly higher than that of CrSeS (7.44%) [28], VS_2_ (6.6%) [20], and TiS_2_ (5.22%) [49]. This corresponds to a significant lattice volume expansion of approximately 22% (Figure 3c). Notably, despite this large lattice expansion, the unit cell parameters of electrochemically intercalated LiCrSe_2_ and deintercalated CrSe_2_ remained in excellent agreement with chemically synthesized LiCrSe_2_ and CrSe_2_ (Tables S2 and S3). Although lithiation involves relatively large volume change, no pronounced mechanical degradation is observed under the cycling conditions investigated. The as‐synthesized CrSe_2_ exhibits an intrinsically broad particle‐size distribution (Figure S5), which is largely preserved after electrode preparation. SEM images collected after 20 cycles show that the particles maintain similar sizes and overall morphology, with no clear evidence of particle fracture or pulverization (Figure S21).

The *in operando* XRD (Figure 4a) revealed only a single structural transition during lithiation which is in line with the DFT investigations. This transition coincides precisely with the voltage inflection from the plateau to the sloping region in the GCD profile, indicating that the structural and electronic evolutions occur simultaneously. The cross‐sectional Scanning Transmission Electron Microscopy analysis of solids extracted from electrodes with the electrochemical experiment halted at approximately x ≈ 0.5 in the Li_0.5_CrSe_2_ phase (Figure S22) provides further insight into the lithiation mechanism. One region displays well‐ordered lattice fringes consistent with pristine CrSe_2_, while adjacent regions exhibit expanded and locally bent lattice planes that can be attributed to the LiCrSe_2_ phase. This observation suggests that lithiation proceeds heterogeneously within a single particle. Combining these observations with the DFT‐calculated density of states for CrSe_2_ and LiCrSe_2_, the process can be rationalized as a progressive conductivity change driven by lithiation. During lithiation, Li intercalates initially at the particle surface, forming a LiCrSe_2_ shell around a metallic CrSe_2_ core. In this stage, corresponding to the plateau region, the metallic core ensures high overall conductivity, consistent with the low and stable interfacial resistance observed by EIS (Figure 3d). As lithiation proceeds, the LiCrSe_2_ shell thickens, and once its semiconducting layer reaches a critical thickness, the overall electronic conductivity of the core‐shell structure decreases sharply. This increase in charge‐transfer resistance manifests as the voltage drop and transition from the plateau to the sloping region. Therefore, we hypothesize that the observed plateau‐slope behavior originates from the dynamic formation of a conductive‐semiconductive core‐shell configuration within CrSe_2_ during lithiation, rather than from multiple discrete phase transitions.

## 3 Conclusions

In summary, combined theoretical and experimental investigations reveal that CrSe_2_ undergoes a single, reversible topotactic intercalation process within the voltage range of 1.0–3.0 V. DFT calculations reveal that Li insertion is limited to octahedral sites, maintaining the *P*‐3*m*1 framework and inducing a transition from metallic to semiconducting behavior upon full lithiation. Experimental XRD and XANES confirm the structural stability and hybrid Cr‐Se redox character of the CrSe_2 –_ LiCrSe_2_ pair. Electrochemical measurements identify a distinct plateau–slope discharge profile, governed by conductivity evolution rather than multiphase transitions. *in operando* XRD reveals only one structural conversion, while EIS and DOS analyses collectively indicate that the transition from plateau to slope arises from the formation and growth of a LiCrSe_2_ shell surrounding a metallic CrSe_2_ core. This core‐shell evolution progressively increases interfacial resistance and triggers the observed voltage slope. The µ^+^SR measurements further confirm fast intrinsic Li^+^ diffusion in LiCrSe_2_, supporting its excellent kinetic properties. Overall, CrSe_2_ exemplifies conductivity‐governed intercalation in layered electrodes, where a gradual conductivity change drives the transition from plateau to slope. The proposed core‐shell evolution offers a plausible explanation for this behavior, providing a foundation for future TMDs material studies aimed at directly resolving and potentially controlling such conductivity‐driven transformations.

Several challenges must still be addressed before CrSe_2_ can be considered for practical applications. First, the synthesis demonstrated here is restricted to laboratory scale, therefore the development of scalable preparation method will be required. LiCrSe_2_ is sensitive to ambient conditions, which indicates that controlled processing or appropriate surface‑stabilization strategies will be necessary for practical electrode fabrication. Nevertheless, the mechanistic insights obtained in this work establish a useful framework for understanding how conductivity changes influence charge‐discharge behavior in Li‐ion cells. This creates a clear route toward rational optimization of the electronic structure in chalcogenide‐based electrodes, especially since density‐functional theory provides an effective way to guide such optimization in a systematic manner through the density of state calculations.

## Funding

A.Y.G. would like to acknowledge the support by EPSRC (EP/W03333X/1) and UKRI (Grant No. 1249). A.G. and J.D. gratefully acknowledge the financial support by the Deutsche Forschungsgemeinschaft (DFG, German Research Foundation) under Germany´s Excellence Strategy‐EXC 2154‐Project number 390874152. [JD8.1]. Furthermore, computer time provided by the state of Baden‐Württemberg through bwHPC and the German Research Foundation through grant no INST 40/575‐1 FUGG (Justus 2 Cluster) and funding by Dr. Barbara Mez‐Starck Stiftung are highly appreciated. This work contributes to the research performed at CELEST (Center for Electrochemical Energy Storage Ulm‐Karlsruhe).

## Conflicts of Interest

The authors declare no conflicts of interest.

## Supporting information




**Supporting File**: advs74574‐sup‐0001‐SuppMat.docx.

## Data Availability

The data that support the findings of this study are openly available in NOMAD repository at https://dx.doi.org/10.17172/NOMAD/2024.07.18‐1, reference number 202407181.

## References

[1] X. Chen , R. Zhan , Z. Chen , et al., “Enhancing Fast‐Charging Capability of Thick Electrode in Lithium‐Ion Batteries Through Electronic/Ionic Hybrid Conductive Additive Engineering,” Advanced Energy Materials 15 (2025): 2500242, 10.1002/aenm.202500242.

[2] C. Y. Wang , T. Liu , X. G. Yang , et al., “Fast Charging of Energy‐Dense Lithium‐Ion Batteries,” Nature 611 (2022): 485–490, 10.1038/s41586-022-05281-0. 36224388

[3] Z. Jin , Q. Cheng , S. T. Bao , et al., “Iterative Synthesis of Contorted Macromolecular Ladders for Fast‐Charging and Long‐Life Lithium Batteries,” Journal of the American Chemical Society 144 (2022): 13973–13980, 10.1021/jacs.2c06527. 35878396

[4] M. Weiss , R. Ruess , J. Kasnatscheew , et al., “Fast Charging of Lithium‐Ion Batteries: A Review of Materials Aspects,” Advanced Energy Materials 11 (2021): 2101126, 10.1002/aenm.202101126.

[5] C. Zhao , Z. Yang , X. Zhou , et al., “Recent Progress on Electrolyte Boosting Initial Coulombic Efficiency in Lithium‐Ion Batteries,” Advanced Functional Materials 34 (2023): 2303457, 10.1002/adfm.202303457.

[6] J. Bi , Z. Du , J. Sun , et al., “On the Road to the Frontiers of Lithium‐Ion Batteries: A Review and Outlook of Graphene Anodes,” Advanced Materials 35 (2023): 2210734, 10.1002/adma.202210734. 36623267

[7] F. M. N. U. Khan , M. G. Rasul , A. S. M. Sayem , and N. Mandal , “Maximizing Energy Density of Lithium‐Ion Batteries for Electric Vehicles: A Critical Review,” Energy Reports 9 (2023): 11–21, 10.1016/j.egyr.2023.08.069.

[8] W. D. Dou , M. T. Zheng , W. Zhang , et al., “Review on the Binders for Sustainable High‐Energy‐Density Lithium Ion Batteries: Status, Solutions, and Prospects,” Advanced Functional Materials 33 (2023): 2305161, 10.1002/adfm.202305161.

[9] Y. K. Liu , C. Z. Zhao , J. Du , X. Q. Zhang , A. B. Chen , and Q. Zhang , “Research Progresses of Liquid Electrolytes in Lithium‐Ion Batteries,” Small 19 (2023): 2205315, 10.1002/smll.202205315. 36470676

[10] T. Dong , S. Zhang , Z. Ren , et al., “Electrolyte Engineering Toward High Performance High Nickel (Ni ≥ 80%) Lithium‐Ion Batteries,” Advanced Science 11 (2024): 2305753, 10.1002/advs.202305753. 38044323 PMC10870087

[11] S. Z. Wang , J. Y. Shi , Z. H. Liu , and Y. Y. Xia , “Advanced Ether‐Based Electrolytes for Lithium‐Ion Batteries,” Advanced Energy Materials 14 (2024): 2401526, 10.1002/aenm.202401526.

[12] K. Brijesh , M. Jareer , G. Lakshmi Sagar , et al., “Advanced Electrolyte Additives for Lithium‐Ion Batteries: Classification, Function, and Future Directions,” Journal of Physical Chemistry C 129 (2025): 11221–11251, 10.1021/acs.jpcc.5c01331.

[13] J. Wang , Y. Liu , Q. Cai , A. Dong , D. Yang , and D. Zhao , “Hierarchically Porous Silica Membrane as Separator for High‐Performance Lithium‐Ion Batteries,” Advanced Materials 34 (2022): 2107957, 10.1002/adma.202107957. 34741777

[14] X. Wu , H. Zhang , J. Zhang , and X. W. D. Lou , “Recent Advances on Transition Metal Dichalcogenides for Electrochemical Energy Conversion,” Advanced Materials 33 (2021): 2008376, 10.1002/adma.202008376. 34405909

[15] G. Chaney , A. Ibrahim , F. Ersan , D. Cakir , and C. Ataca , “Comprehensive Study of Lithium Adsorption and Diffusion on Janus Mo/WXY (X, Y = S, Se, Te) Using First‐Principles and Machine Learning Approaches,” ACS Applied Materials & Interfaces 13 (2021): 36388–36406, 10.1021/acsami.1c05508. 34304560

[16] H. L. Wong , M. D. Hossain , M. Y. Xu , et al., “High Lithiophilicity and Li Diffusion Rate on 1T Phase Transition Metal Dichalcogenides as Effective Li Regulating Materials for Dendrite‐Free Metal Anodes,” Journal of Materials Chemistry A 12 (2024): 23810–23818, 10.1039/d4ta03686g.

[17] Q. Zhang , S. Tan , R. G. Mendes , et al., “Extremely Weak van der Waals Coupling in Vertical ReS_2_ Nanowalls for High‐Current‐Density Lithium‐Ion Batteries,” Advanced Materials 28 (2016): 2616–2623, 10.1002/adma.201505498. 26822853

[18] S. Fleischmann , H. Shao , P. L. Taberna , P. Rozier , and P. Simon , “Electrochemically Induced Deformation Determines the Rate of Lithium Intercalation in Bulk TiS_2_ ,” ACS Energy Letters 6 (2021): 4173–4178, 10.1021/acsenergylett.1c01934.

[19] P. Li , X. B. Zheng , H. X. Yu , et al., “Electrochemical Potassium/Lithium‐Ion Intercalation into TiSe_2_: Kinetics and Mechanism,” Energy Storage Materials 16 (2019): 512–518, 10.1016/j.ensm.2018.09.014.

[20] X. Zhang , Q. He , X. M. Xu , et al., “Insights into the Storage Mechanism of Layered VS_2_ Cathode in Alkali Metal‐Ion Batteries,” Advanced Energy Materials 10 (2020): 1904118, 10.1002/aenm.201904118.

[21] Z. Wu , C. Lu , Y. Wang , et al., “Ultrathin VSe_2_ Nanosheets with Fast Ion Diffusion and Robust Structural Stability for Rechargeable Zinc‐Ion Battery Cathode,” Small 16 (2020): 2000698, 10.1002/smll.202000698. 32776405

[22] S. Kim , Y. J. Kim , and W. H. Ryu , “Zirconium Disulfides as an Electrode Material Alternative for Li‐Ion Batteries,” Applied Surface Science 547 (2021): 149029, 10.1016/j.apsusc.2021.149029.

[23] Y. Onuki , R. Inada , S. Tanuma , S. Yamanaka , and H. Kamimura , “Electrochemical Characteristics of ZrSe_2_ in a Secondary Lithium Battery,” Solid State Ionics 8 (1983): 141–145, 10.1016/0167-2738(83)90075-9.

[24] D. S. Fan , Y. Wang , X. D. Zhao , et al., “A Novel NASICON‐Na_3.4_MnV_0.2_Cr_0.2_Ti_0.6_(PO_4_)_3_ Cathode with Ultrahigh Energy Density and Remarkable Cycling Stability Toward Practical Na‐Ion Batteries,” Materials Today 86 (2025): 63–73, 10.1016/j.mattod.2025.03.010.

[25] G. Hautier , A. Jain , S. P. Ong , et al., “Phosphates as Lithium‐Ion Battery Cathodes: An Evaluation Based on High‐Throughput ab Initio Calculations,” Chemistry of Materials 23 (2011): 3495–3508, 10.1021/cm200949v.

[26] S. J. Clark , D. Wang , A. R. Armstrong , and P. G. Bruce , “Li(V_0.5_Ti_0.5_)S_2_ as a 1 V Lithium Intercalation Electrode,” Nature Communications 7 (2016): 10898, 10.1038/ncomms10898. PMC4802118 26996753

[27] B. L. Hoff , J. M. Moya , F. Yuan , et al., “Chemical Exfoliation for the Preparation of CrSe_2_ Nanoribbons and CrTe_2–x_ Nanosheets,” Chemistry of Materials 37 (2025): 5333–5343, 10.1021/acs.chemmater.5c01115.

[28] S. Y. Yang , D. R. Shi , T. Wang , et al., “High‐Rate Cathode CrSSe Based on Anion Reactions for Lithium‐Ion Batteries,” Journal of Materials Chemistry A 8 (2020): 25739–25745, 10.1039/d0ta08012h.

[29] B. Zhao , D. Y. Shen , Z. C. Zhang , et al., “2D Metallic Transition‐Metal Dichalcogenides: Structures, Synthesis, Properties, and Applications,” Advanced Functional Materials 31 (2021): 2105132, 10.1002/adfm.202105132.

[30] S. Susarla , A. Kutana , J. A. Hachtel , et al., “Quaternary 2D Transition Metal Dichalcogenides (TMDs) with Tunable Bandgap,” Advanced Materials 29 (2017): 1702457, 10.1002/adma.201702457. 28707411

[31] A. Chaves , J. G. Azadani , H. Alsalman , et al., “Bandgap Engineering of Two‐Dimensional Semiconductor Materials,” NPJ 2D Materials and Applications 4 (2020): 29, 10.1038/s41699-020-00162-4.

[32] S. K. Geng , H. Wang , K. Y. Xu , et al., “Improve the Capacity of MoS_2_ for Aqueous Zinc Ion Batteries by Regulating the Electron Spin States of Mo via Se Doping,” ACS Applied Energy Materials 8 (2025): 12904–12911, 10.1021/acsaem.5c02116.

[33] M. Sotoudeh and A. Gross , “Descriptor and Scaling Relations for Ion Mobility in Crystalline Solids,” JACS Au 2 (2022): 463–471, 10.1021/jacsau.1c00505. 35252995 PMC8889558

[34] M. Sotoudeh , S. Baumgart , M. Dillenz , et al., “Ion Mobility in Crystalline Battery Materials,” ChemRxiv (2023): 2bb18, 10.26434/chemrxiv-2023-2bb18.

[35] E. Nocerino , S. Kobayashi , C. Witteveen , et al., “Competition Between Magnetic Interactions and Structural Instabilities Leading to Itinerant Frustration in the Triangular Lattice Antiferromagnet LiCrSe_2_ ,” Communications Materials 4 (2023): 81, 10.1038/s43246-023-00407-x.

[36] W. Li , J. Döhn , J. Chen , et al., “Reversible K‐Ion Intercalation in CrSe_2_ Cathodes for Potassium‐Ion Batteries: Combined Operando PXRD and DFT Studies,” Journal of Materials Chemistry A 12 (2024): 31276–31283, 10.1039/d4ta05114a.

[37] H. Euchner and A. Groß , “Atomistic Modeling of Li‐ and Post‐Li‐Ion Batteries,” Physical Review Materials 6 (2022): 040302, 10.1103/PhysRevMaterials.6.040302.

[38] W. Li , N. Wolff , A. K. Samuel , et al., “Unlocking High‐Performance Supercapacitor Behavior and Sustained Chemical Stability of 2D Metallic CrSe_2_ by Optimal Electrolyte Selection,” Chemelectrochem 10 (2023): 202300428, 10.1002/celc.202300428.

[39] F. M. Engelsman , G. A. Wiegers , F. Jellinek , and B. Vanlaar , “Crystal Structures and Magnetic Structures of Some Metal(I) Chromium(III) Sulfides and Selenides,” Journal of Solid State Chemistry 6 (1973): 574–582, 10.1016/S0022-4596(73)80018-0.

[40] C. F. van Bruggen , R. J. Haange , G. A. Wiegers , and D. K. G. de Boer , “CrSe_2_, a New Layered Dichalcogenide,” Physica B+C 99 (1980): 166–172, 10.1016/0378-4363(80)90226-0.

[41] J. O. Ticknor , I. Umegaki , R. M. L. McFadden , et al., “Investigation of Ionic and Anomalous Magnetic Behavior in CrSe_2_ Using 8Li β‐NMR,” RSC Advances 10 (2020): 8190–8197, 10.1039/c9ra07065f. 35497818 PMC9049877

[42] Y. Kim , K. Park , S. Song , J. Han , and J. B. Goodenough , “Access to M^3^ + / M^2^ + Redox Couples in Layered LiMS_2_ Sulfides (M = Ti , V , Cr ) as Anodes for Li‐Ion Battery,” Journal of the Electrochemical Society 156 (2009): A703, 10.1149/1.3151856.

[43] A. Pantelouris , H. Modrow , M. Pantelouris , J. Hormes , and D. Reinen , “The Influence of Coordination Geometry and Valency on the K‐Edge Absorption Near Edge Spectra of Selected Chromium Compounds,” Chemical Physics 300 (2004): 13–22, 10.1016/j.chemphys.2003.12.017.

[44] S. Kobayashi , N. Katayama , T. Manjo , et al., “Linear Trimer Formation with Antiferromagnetic Ordering in 1*T*‐CrSe_2_ Originating from Peierls‐like Instabilities and Interlayer Se–Se Interactions,” Inorganic Chemistry 58 (2019): 14304–14315, 10.1021/acs.inorgchem.9b00186. 30964663

[45] M. Amores , T. E. Ashton , P. J. Baker , E. J. Cussen , and S. A. Corr , “Fast Microwave‐Assisted Synthesis of Li‐Stuffed Garnets and Insights into Li Diffusion from Muon Spin Spectroscopy,” Journal of Materials Chemistry A 4 (2016): 1729–1736, 10.1039/c5ta08107f.

[46] I. McClelland , B. Johnston , P. J. Baker , M. Amores , E. J. Cussen , and S. A. Corr , “Muon Spectroscopy for Investigating Diffusion in Energy Storage Materials,” Annual Review of Materials Research 50 (2020): 371–393, 10.1146/annurev-matsci-110519-110507.

[47] J. Sugiyama , K. Mukai , Y. Ikedo , H. Nozaki , M. Mansson , and I. Watanabe , “Li Diffusion in LixCoO_2_ Probed by Muon‐Spin Spectroscopy,” Physical Review Letters 103 (2009): 147601, 10.1103/PhysRevLett.103.147601. 19905603

[48] I. D. Johnson , T. E. Ashton , E. Blagovidova , et al., “Mechanistic Insights of Li+ Diffusion Within Doped LiFePO_4_ from Muon Spectroscopy,” Scientific Reports 8 (2018): 4114, 10.1038/s41598-018-22435-1. 29515155 PMC5841297

[49] M. Månsson , H. Nozaki , J. M. Wikberg , et al., “Lithium Diffusion & Magnetism in Battery Cathode Material Li_x_Ni_1/3_Co_1/3_Mn_1/3_O_2_ ,” Journal of Physics: Conference Series 551 (2014): 012037, 10.1088/1742-6596/551/1/012037.

[50] P. Vadhva , J. Hu , M. J. Johnson , et al., “Electrochemical Impedance Spectroscopy for All‐Solid‐State Batteries: Theory, Methods and Future Outlook,” Chemelectrochem 8 (2021): 1930–1947, 10.1002/celc.202100108.

