# Supplementary material for: Lithiation‐Driven LiCrSe2 Shell Growth on Metallic CrSe2 Core Governs the Plateau–Slope Behavior
Source: Adv Sci (Weinh). 2026 Feb 27;13(26):e23702. doi: 10.1002/advs.202523702 (PMC13159162; doi:10.1002/advs.202523702)
Supplement: Supplementary file 1 — Supporting File: advs74574‐sup‐0001‐SuppMat.docx. [file ADVS-13-e23702-s001.docx]

Supporting Information

Lithiation-Driven LiCrSe_2_ Shell Growth on Metallic CrSe_2_ Core Governs the Plateau–Slope Behavior

Weihao Li, Johannes Döhn, Xiao Han, Licheng Zhang, Dave M. Pickup, Xiong Xiao, Yidong Miao, Arun Kumar Samuel, Emily R. Draper, Alan V. Chadwick, Stephen Sproules, Stephen Cottrell, Alan J. Drew, Changhua An, Axel Gross, Alexey Y. Ganin*

**Experimental section**

**Material preparation**

Synthesis of graphite-added LiCrSe_2_ was carried out on a 400 mg scale with handling of the reagents and products inside an argon-filled glove box (MBraun, <0.1 ppm of O_2_). Stoichiometric amounts of Cr powder (Alfa Aesar, 99.94 %, -200 mesh, metal basis), Se shots (Alfa Aesar, 99.99 %, metal basis) and 10 wt.% graphite powder (Alfa Aesar, -100 mesh, 99.9995 %, metal basis) were loaded into a Pyrex insert together with Li metal (Sigma-Aldrich, 99.9 %, metal basis). The insert was placed inside a Pyrex ampoule and sealed under vacuum with a blow torch. The ampoule was then placed upright in a muffle box furnace (Lenton) and heated at 250°C (1°C min^–1^ ramp) for 4 hours. The temperature was then raised to 600°C (1°C min^–1^ heating ramp, 72 h dwell, 5°C min^–1^ cooling). The sample was then taken out of the ampoule, ground using mortar and pestle, sealed under vacuum again and further reannealed at 600°C (1°C min^–1^ heating ramp, 48 h dwell, 5°C min^–1^ cooling ramp).

Graphite-added CrSe_2_ were prepared by the deintercalation of graphite-added LiCrSe_2_ powders with iodine in acetonitrile solution respectively. Typically, inside an argon-filled glove box (MBraun, <0.1 ppm of O_2_), 200 mg of Graphite-added LiCrSe_2_ powder was loaded into a round bottom flask (equipped with a stirring bar). The flask was attached to a Schlenk line and ca. 4 mL (given in excess) of 0.5 M iodine in acetonitrile solution (Alfa Aesar, anhydrous, 99.8 %) was added under N_2_ flow. The mixture was stirred 24 hours at ambient temperature (ca. 20°C). The product was then filtered in air resulting in a black powder on the filter. The powder was washed with 100 ml of acetonitrile, then with excess of deionized water, finally rinsed with 100 ml of ethanol and left to dry overnight in an evacuated desiccator.

**Electrochemical characterization**

The Graphite-added CrSe_2_ active material was mixed with PVDF binder (Sigma-Aldrich, Mw~634000) and Super P carbon black (CB, Alfa Aesar, ≥ 99%) in a weight ratio of 80:10:10 and dissolved in NMP (Sigma-Aldrich, ≥ 99%). The mixture was added into ball milling jar which was sealed under argon atmosphere and carried out at room temperature at frequency 20 s^–1^. The mixture was ground thoroughly until a uniform black slurry was formed. The slurry was coated onto aluminum foil using a 20 μm coater and dried at 60 °C overnight. Disks with an area of ~1.057 cm^2^ (diameter of 1.16 cm) were punched from the foil corresponding to the average loading of the mixture on the foil at 1.14 mg cm^‒2^ (corresponding to 0.912 mg cm^‒2^ of the active CrSe_2_). A Li foil (Sigma-Aldrich, 99.9 %, metal basis) disk with the area of ~1.057 cm^2^ (diameter 1.16 cm, thickness 0.38 mm) served as an anode.

1M LiPF6 in ethylene carbonate and dimethyl carbonate, 1.0 M LiPF_6_ in EC/DMC=50/50 (v/v) (Sigma-Aldrich, battery grade) was applied as electrolyte.

Battery tests were carried out in two types of battery cells. Swagelok cells were routinely used for all experiments except for *in operando* XRD studies as discussed below. The cells were assembled by sandwiching glass fiber membrane (Whatman GF/A, diameter 13.94 mm, thickness 0.26 mm) wetted with 60 μL of 1 M LiPF_6_ EC/DMC solution between two electrodes. Cells were assembled following the order of anode shell, spring, current collector, Lithium disk anode, separator, soaking electrolyte, cathode disk and then cathode shell. For the *in operando* XRD studies, the CR2032 type cell with the identical cathode, separator and Li-metal anode was used but with a window made from Kapton on the cathode side to allow the beam transmittance. Cyclic voltammetry (CV) and galvanostatic charge-discharge (GCD) techniques were conducted on a Biologic, SP-150 (EC-labs) potentiostat and a Lanhe battery cycler, respectively.

**Material** **characterization**

PXRD of non-air-sensitive samples were measured on a Rigaku MiniFlex 6G diffractometer (CuK_α1_ and CuK_α2_ wavelengths - 1.5406 and 1.5444 Å respectively) equipped with a D/teX Ultra detector operating in the Bragg–Brentano geometry. Powder samples were carefully packed onto zero background holders and levelled using a glass microscope slide. SC electrodes were also tested by attaching them directly to the holder with a double-sided tape. Diffraction patterns were collected with a step size of 0.015° and time per step of 1° min^–1^. The sample holder was spun during the measurements at 10 rpm.

PXRD measurements of air-sensitive samples, LiCrSe_2_ was performed on a PANalytical Empyrean diffractometer (CuK_α1_ and CuK_α2_ wavelengths - 1.5406 and 1.5444 Å respectively) operating in the Debye-Scherrer geometry. The samples were packed inside an Ar-filled glove box (MBraun, <0.1 ppm O_2_) into 0.5 mm diameter (0.1 mm wall thickness) No.50 special glass capillaries (Hilgenberg), which were sealed with a blow torch. The PXRD patterns were collected with a step size of 0.016° and time per step of 5° min^–1^.

Le Bail refinement of selected PXRD data was performed using GSAS-II software. ^[1]^ The refined parameters included unit cell parameters, sample displacement, strain, and crystallite broadening. Background was fitted using shifted Chebyshev polynomic shape.

*In operando* XRD measurement was performed on a one-side open CR2032 coincell. The XRD experiment was carried out on PANalytical Empyrean diffractometer with Cu Kα radiation (λ=0.154 nm), in 2θ ranges from 26° to 35° with a step size of ~ 0.03° and an overall time of about 15 min per scan. The *in operando* cell was assembled using the identical electrode composition for standard electrochemical tests.

According to the crystallography theory for *P*-3*m*1 space group, (002) peaks directly reflect changes along c axes of the layered structure, where (101) peaks can deduce *a* through formula (1) for hexagonal system which can be converted as formula (2)

| $\frac{1}{d_{hkl}^{2}}=\frac{4}{3} \left( \frac{h^{2}+hk+k^{2}}{a^{2}} \right) + \frac{l^{2}}{c^{2}}$ | (1) |
| --- | --- |
| $\frac{1}{d_{101}^{2}}= \frac{4}{3a^{2}}+ \frac{1}{c^{2}}$ | (2) |

Microscopy studies: For Scanning electron microscope/Energy Dispersive X-Ray (SEM / EDX) studies a small amount of sample powders was attached to a sticky carbon tape, which was shaken to remove excess powder. The tape was attached to an Al holder. Morphology studies were carried out on a Scanning Electron Microscope (TESCAN CLARA) equipped with a Field Emission Gun electron source which was coupled with an Oxford Instruments UltimMax 65 with an Aztec live interface EDX system for elemental analysis.

TEM images were performed on a Talos F200 X high-resolution field-emission TEM (FEI) with acceleration voltage of 200 kV. The samples were ultrasonically dispersed in ethanol, deposited onto ultrathin-thin carbon film copper grids (Beijing XXBR Technology), and dried under an infrared lamp prior to imaging

CrSe_2_ particles were extracted from electrodes galvanostatically discharged at 0.1 C to half of the theoretical intercalation capacity, which is expected to correspond to approximately half of the lithium insertion. Coin cells were disassembled using an MIT coin-cell press equipped with a disassembly crimp. The recovered electrodes were rinsed with an ethylene carbonate/dimethyl carbonate (EC/DMC, 1:1 v/v) solvent mixture inside an argon-filled glovebox to remove residual electrolyte.

Cross-sectional specimens for transmission electron microscopy (TEM) characterization were prepared using a focused ion beam–scanning electron microscope (FIB–SEM, FEI Helios NanoLab 600i) operated at accelerating voltages between 2 and 30 kV. To minimize contamination from moisture and oxygen, samples were transferred from the argon-filled glovebox to the FIB–SEM in a sealed container, limiting air exposure to less than 3 min. To reduce damage induced by the high-energy Ga^+^ ion beam during sample preparation, an approximately 500 nm thick Pt/C protective layer was first deposited on the surface of the Li_X_CrSe_2_ (x ~ 0.5) particles using electron-beam-assisted deposition, followed by an additional approximately 2 μm thick Pt/C layer deposited using ion-beam-assisted deposition. The lamellae were subsequently thinned to a thickness below 150 nm and cleaned using low-voltage Ga⁺ ion milling (2–5 kV), followed by plasma treatment.

High-resolution annular bright-field (ABF) and high-angle annular dark-field (HAADF) scanning TEM (STEM) imaging, together with energy-dispersive X-ray spectroscopy (EDS) elemental mapping, were performed using a double spherical aberration-corrected transmission electron microscope (JEM-ARM300F2, JEOL).

Cr and Se K-edge X-ray absorption spectroscopy (XAS) data were collected at beamline B18 at the Diamond Light Source, U.K. Samples were diluted with crystalline cellulose and pressed into a 13 mm diameter pellets using a hydraulic press. Incident X-ray wavelengths were selected using a Si(111) double-crystal monochromator. Extended X-ray absorption fine structure (EXAFS) spectra and X-ray absorption near-edge structure (XANES) spectra were collected in transmission mode using two gas-filled ionization chambers. In the case of the Cr K-edge measurements, a Cr metal foil was placed in front of a third ionization chamber in order to correct for instrumental drift. For the Se K-edge measurements, a Se foil was not available; instead, a Pt metal foil was used since the Pt L2-edge energy of 13.2726 keV is close to the Se K-edge energy of 12.6578 keV. Initial processing of the data, normalization and background removal, was performed using the program ATHENA.^[2]^ The k3-weighted EXAFS spectra were modelled using the program ARTEMIS [Ravel] to determine interatomic Cr-Se, Cr-Cr and Se-Se distances within each sample.

The resistivity measurements on LiCrSe_2_ and CrSe_2_ were carried out inside an Ar-filled glove box. In a typical experiment, 1.5 g of powder was was compressed to a thickness of approximately 2 mm (diameter 12.7 mm) under a pressure of ~ 395 kg cm^–2^. The electrical resistance was recorded under constant pressure using a BioLogic SP-150 potentiostat (EC-Lab). The cell was heated with a help of a heating tape with 3 points recorded at 288 K, 318 K and 338 K respectively.

The µ^+^SR experiments were conducted at the ISIS pulsed muon and neutron source on the EMU instrument ^[3]^. These samples were prepared for analysis by transferring *ca*. 1 g into a standard ISIS Ti powder cell with a Ti foil window. Ti was chosen as a sample holder material because it has negligible internal magnetic fields and therefore gave a simple background feature which could be easily subtracted in the analysis. The Li-ion hopping rates are extracted from the μ^+^SR data, analyzed by Mantid Muon Analysis package.^[4]^ The ZF and the two LF (5 G and 10 G) spectra are well fitted by a combination of the dynamic Kubo-Toyabe (KT) function multiplied by a simple exponential relaxation and a non-relaxing background (BG) signal as a result of a fraction of muons stopped in the silver plate mask on the sample holder. The ZF, 5 G LF, and 10 G LF spectra were fitted with the function as formula (3):

| $A(t)=A_{KT}G^{DGKT}\left( \Delta,\nu,t,H_{LF} \right)\times\exp\left( -\lambda_{KT}t \right)+A_{BG}$ | (3) |
| --- | --- |
|  |  |

The parameters $A_{KT}$ and $A_{BG}$ represent the asymmetries of their respective components. $\Delta$ and $\nu$correspond to the width of the local field distribution and the field fluctuation rate at the muon site, respectively. Under the conditions $\nu=0$ and $H_{LF}=0$, the $G^{DGKT}\left( \Delta,\nu,t,H_{LF} \right)$ reduces to the sample static Gaussian KT function at ZF. Figure S7 displays the ZF and LF µ^+^SR time spectra collected at 50 K and 200 K.A more detailed of muon data analysis of the high-temperature ion diffusion can be found in ^[5]^

**DFT calculations**

Total energy calculations based on density functional theory (DFT) were performed as implemented in the Vienna Ab Initio Simulation Package.^[6]^ In our investigations we addressed the Li-2s, Cr-3p^6^4s^1^3d^5^, and Se-2s^2^2p^4^ electrons explicitly whereas the remaining ionic cores were treated with the projector augmented wave method.^[7]^ Electronic wave functions were expanded up to energies of 600 eV and it was made sure that total energies converged within a few meV/atom concerning the number of k-points in the first Brillouin zone. All structures were fully relaxed without restricting any internal degree of freedom until all entries of the stress tensor and the forces on each ion were smaller than 0.01 eV/Å. Zero-point energies and entropic contributions were neglected for all energetic considerations.

At room temperature, CrSe_2_ and LiCrSe_2_ are both known to crystallize in space group *P*-3*m*1 with 3 and 4 atoms in the unit cell, respectively. To study the applicability of different DFT functionals, a benchmark on the structural parameters was performed for both unit cells including the widely used exchange-correlation functional suggested by Perdew, Burke, and Ernzerhof (PBE),^[8]^ its optimized version for solids (PBEsol),^[9]^ and the strongly constrained and appropriately normed (SCAN) meta-generalized gradient approximation^[10]^ (Table S1). Additionally, empirical Van-der-Waals corrections were tested for all functionals.^[11]^

For CrSe_2_ all functionals overestimate the lattice constant a while only PBEsol and PBEsol+D3 result in a deviation of less than 0.1 Å from the experimental value. In contrast to that the layer spacing c is only reproduced by PBE and slightly underestimated by SCAN. The PBEsol and PBEsol+D3 predictions underestimate the experimental value for the layer spacing by over 0.3 Å and 0.4 Å. In the case of LiCrSe_2_ all functionals agree very well with the experimental lattice constantly a deviating by at most 0.047 Å in the PBE calculation. The layer spacing c is underestimated by all functionals although not as strongly pronounced as in the case of CrSe_2_. The PBE and SCAN functionals still result in acceptable values with less than 0.1 Å deviation from the experimental value. Although layered dichalcogenides are commonly characterized by weak interplanar coupling,^[12]^ it is remarkable that the addition of corrections for the Van-der-Waals interactions into the calculations increase the underestimation of the layer spacing c in all six cases. Most outstandingly the prediction of the PBE functional agrees almost perfectly with the experimental value of the c-constant in CrSe_2_ whereas it deviates by more than 0.4 Å in the PBE+D3 calculation.

According to the previous discussion, the PBEsol functional and the inclusion of corrections for the Van-der-Waals interactions were considered as non-suitable for the given systems and the PBE and SCAN functionals were chosen for the investigation. As described below in greater detail, PBE was used for most of the geometry optimizations displayed below in Figures S1 and S2, whereas the more computationally expensive SCAN functional was used for certain samples only, displayed in the convex hull in Figure 1b in the main text. The density of states was determined by employing the hybrid HSE06 functional.^[13]^

The PBE geometry relaxations were performed on 628 different configurations with supercells up to 48 atoms in the symmetries *P*-3*m*1, *C*2/*m* and *R*-3*m* - similar to a previous study.^[14]^ For *P*-3*m*1 we considered octahedral and tetrahedral Li occupation. The energy above hull (Figure S2), a computational measure for thermodynamic stability,^[15]^ was evaluated for all configurations following a previously used method.^[16]^

The configurations which had turned out to be energetically most favorable were reassessed with the SCAN functional to obtain more reliable results. After having performed the SCAN geometry optimizations, we calculated the formation energy E_f_ with respect to the fully lithiated (x=1) and delithiated (x=0) states as formula (4):

| $E_{f}=E_{{Li}_{x}Cr{Se}_{2}}-x E_{LiCr{Se}_{2}}-\left( 1-x \right)E_{Cr{Se}_{2}}$ | (4) |
| --- | --- |

where E_conf_ is the energy of the respective configuration to construct the convex hull curve (Figure 1b).^[17]^ The voltage curve (Figure 1c) was constructed according to formula (5):

| $OCV\left( x,x+\Delta x \right)= \frac{E_{{Li}_{x}Cr{Se}_{2}}+ \Delta x E_{Li}- E_{{Li}_{x+\Delta x}Cr{Se}_{2}}}{e \Delta x}$. | (5) |
| --- | --- |

The barriers for Li-diﬀusion were investigated via the nudged elastic band (NEB) method^[17]^ and the SCAN functional applied to a 2x2x2 supercell in the high vacancy limit and the low vacancy limit. As the configurations are fully symmetric only half the diffusion path, i.e., from octahedral to tetrahedral site, had to be considered. Each path was modelled with six distinct intermediate images between. Optimized NEB algorithms were used to optimize the ion positions until the forces on every ion were lower than 0.05 eV Å^−1^.^[18]^ The climbing image method was used to ensure that the transition state was found.^[19]^

One further remark: LiCrSe_2_ has been shown to exhibit a complex magnetic ground state in which the magnetic moments are periodically suppressed and change direction.^[20]^ The precise theoretical treatment of such complex magnetic structures is labor intensive and requires rather elaborate modelling. However, as the chemical and electrochemical properties of interest are mostly determined by differences of energies and the exact magnetic state is rather unimportant for the presented study, we circumvented the modulation of the exact magnetic state and only tested the most common magnetic orderings, i.e., non-magnetic, ferromagnetic, and antiferromagnetic (A-type, C-type, E-type, G-type), in a 2x2x2 supercell of CrSe_2_ and LiCrSe_2_. For both compounds respectively, the resulting total energies corresponding to the different magnetic states were entirely in a range of 15 meV/atom to each other, only the non-magnetic state resulting in a configuration which is energetically less favorable by over 100 meV/atom. Similar results have been obtained in a recent study on monolayer LiCrSe_2_.^[21]^ Furthermore, we found that the C-type, E-type, and G-type states overestimate the lattice spacing c of CrSe_2_ by more than 1 Å compared to the experimental value. In accordance with the just presented results, we assume that respective errors cancel out in the energy differences that were used for the analysis and that the thermodynamic properties of the system are sufficiently well described through the ferromagnetic state. Consequently, we performed spin polarized calculations with ferromagnetic initialization throughout the entire study.

Table S1. DFT Benchmark on the structural parameters including six different functionals for CrSe_2_ and LiCrSe_2_.

|  | **CrSe_2_** | | **LiCrSe_2_** | |
| --- | --- | --- | --- | --- |
|  | ***a* (Å)** | ***c* (Å)** | ***a* (Å)** | ***c* (Å)** |
| **Experiment** | **3.389** | **5.917** | **3.657** | **6.319** |
| **PBE** | **3.502** | **5.933** | **3.706** | **6.244** |
| **PBE + D3** | **3.517** | **5.513** | **3.677** | **6.108** |
| **PBEsol** | **3.440** | **5.590** | **3.652** | **6.141** |
| **PBEsol + D3** | **3.419** | **5.449** | **3.627** | **6.046** |
| **SCAN** | **3.532** | **5.775** | **3.678** | **6.266** |
| **SCAN + rVV10** | **3.523** | **5.698** | **3.670** | **6.231** |


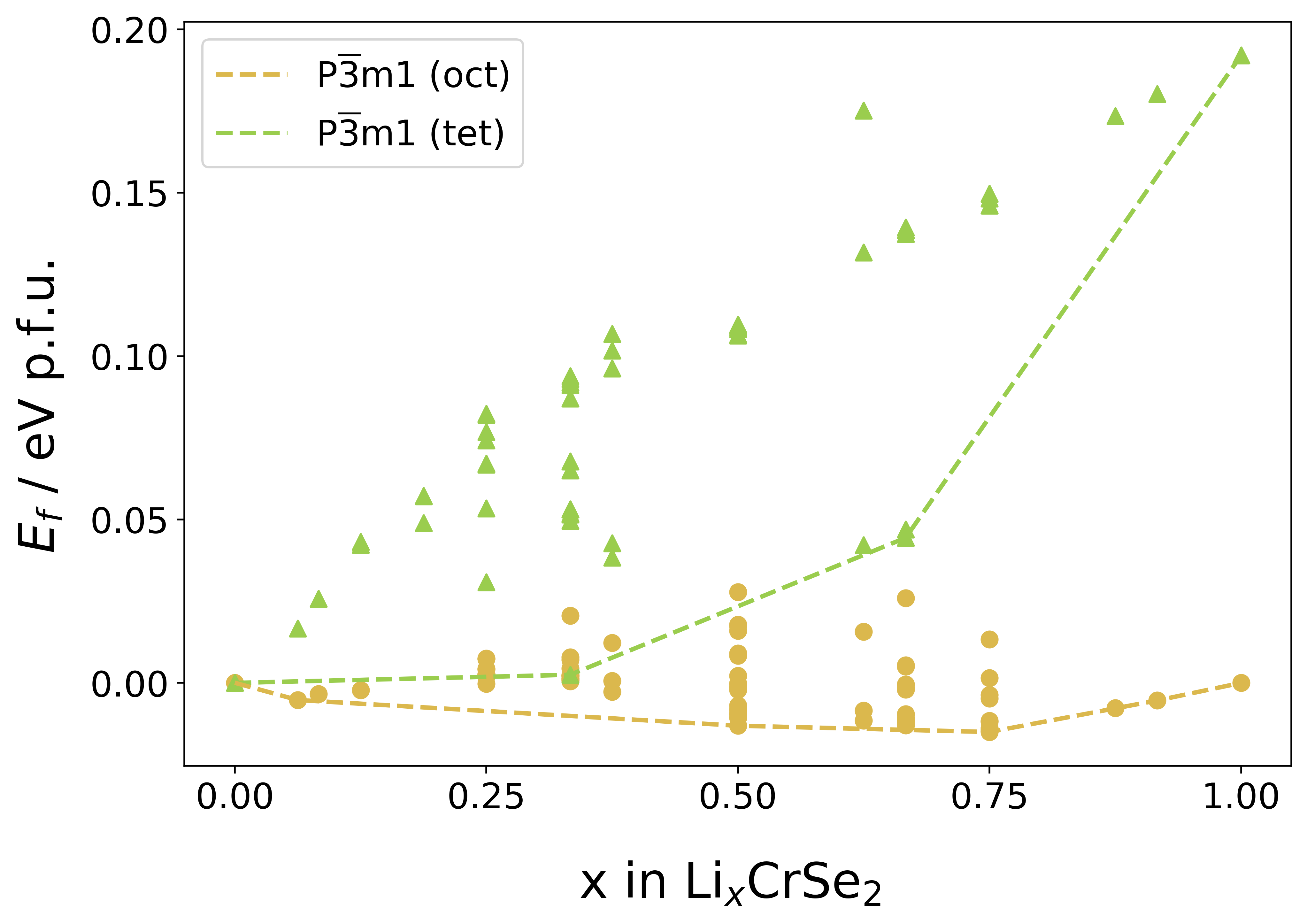


Figure S1. Formation energies of Li_x_CrSe_2_ evaluated with the SCAN functional showing that Li prefers octahedral over tetrahedral coordination.


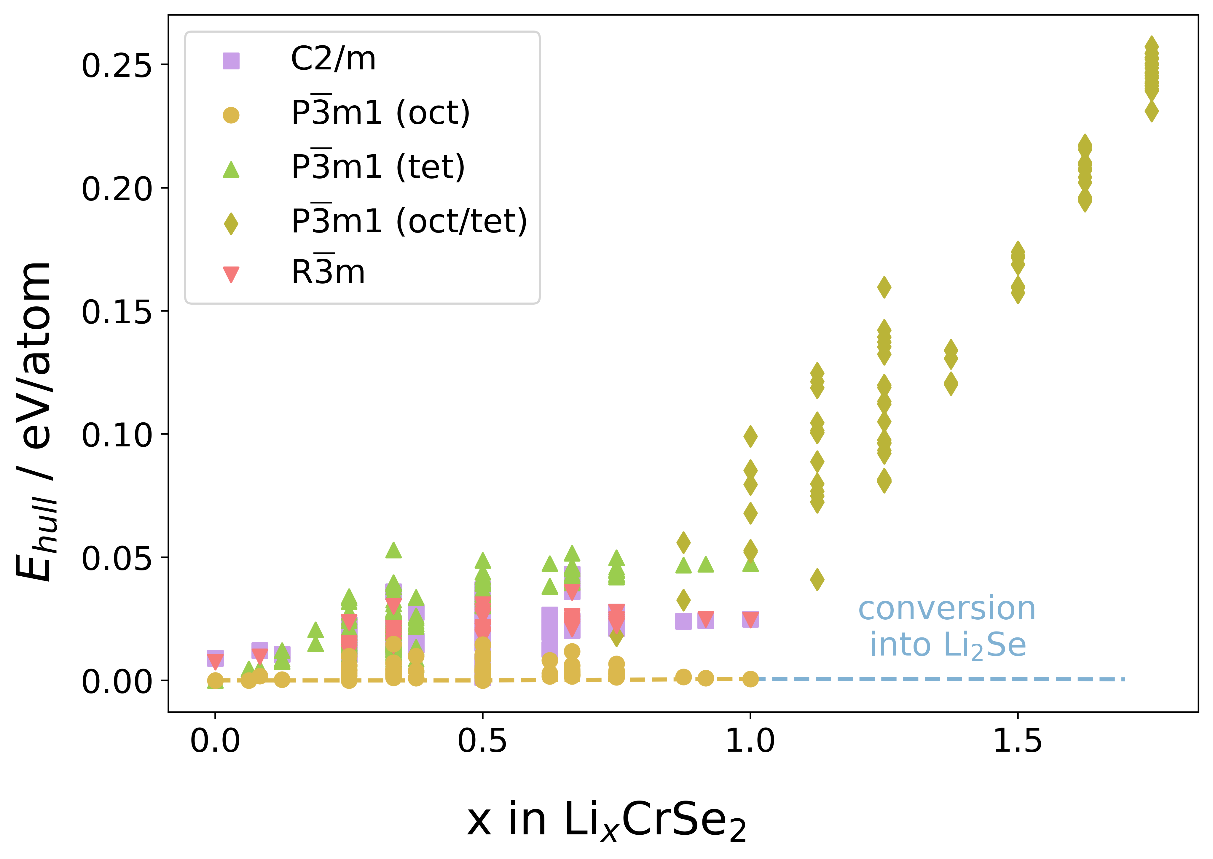


Figure S2. Energy above hull E_hull_ for over 600 configurations Li_x_CrSe_2_ evaluated with the PBE functional. For 0<x<1.0 stable *P*-3*m*1 phases are found, for x>1.0 the conversion into Li_2_Se is energetically much more favorable.


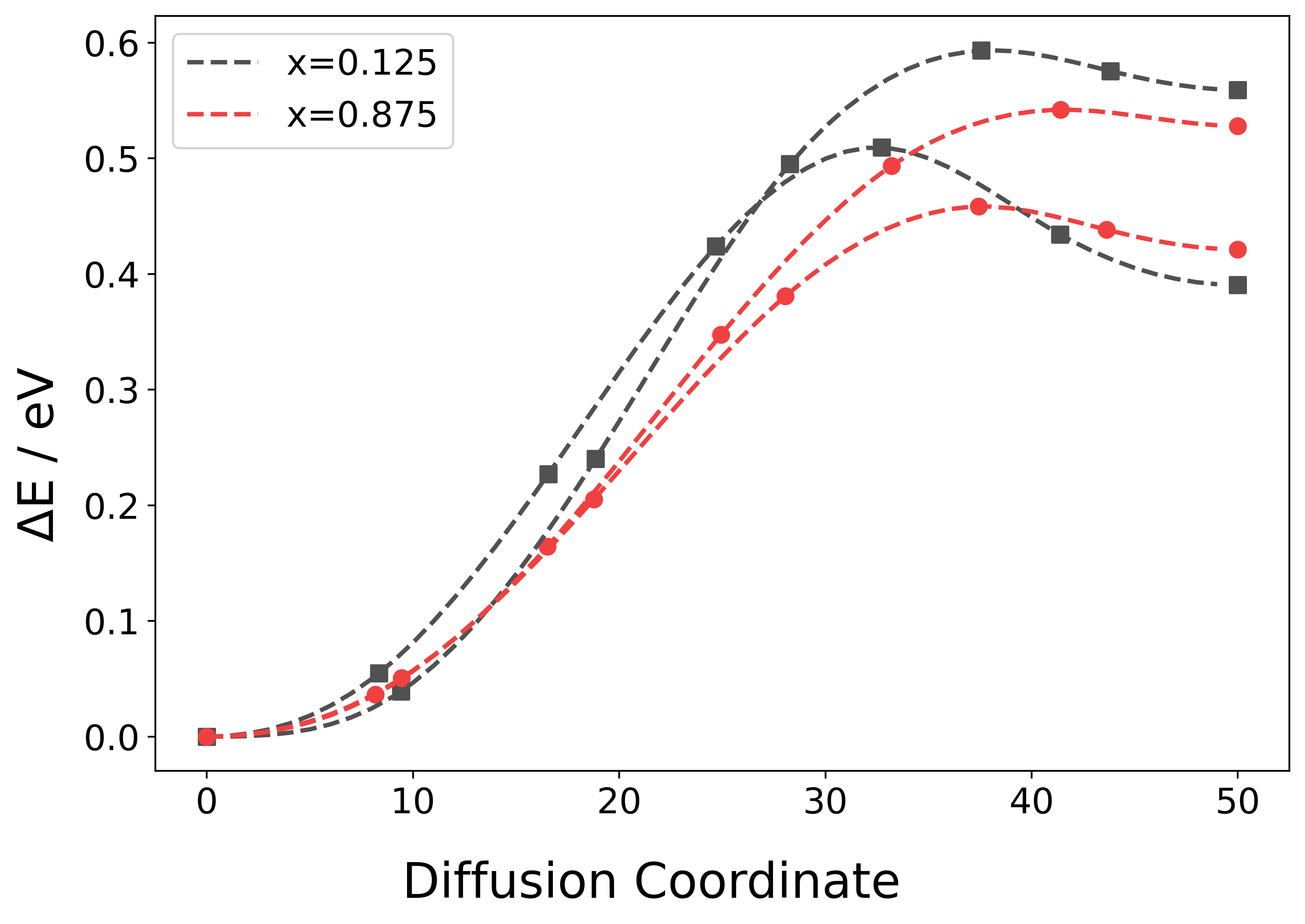


Figure S3 Li-ion energy barriers of Li_X_CrSSe for low (x = 0.125) and high (x = 0.875) lithium-vacancy phases.

Table S2. The summary of cell parameters of the LiCrSe_2_ products and graphite determined by LeBail refinement of the experimental profile.

|  | *a*, Å | *c*, Å | V, Å^3^ |  |
| --- | --- | --- | --- | --- |
| Graphite-added LiCrSe_2_ | 3.65127(5) | 6.29616(10) | 72.6932(11) | This work |
| Graphite | 2.45956(1) | 6.69910(6) | 35.0960(2) | This work |
| LiCrSe_2_ | 3.65328(9) | 6.28535(8) |  | ^[22]^ |

Table S3. The summary of cell parameter of the CrSe_2_ products and graphite determined by LeBail refinement of the experimental profile against previously reported structural model for CrSe_2_. The standard deviations are given in parentheses.

|  | *a*, Å | *c*, Å | V, Å^3^ |  |
| --- | --- | --- | --- | --- |
| Graphite-added CrSe_2_ | 3.3945(6) | 5.9168(10) | 59.044(14) | This work |
| Graphite | 2.4601(9) | 6.6998(6) | 35.115(16) | This work |
| CrSe_2_ | 3.39307(4) | 5.91301(7) |  | ^[22]^ |
| Graphite-added CrSe_2_ | 3.3886(3) | 5.9172(3) |  | ^[23]^ |
| CrSe_2_ | 3.3898(3) | 5.9099(4) |  | ^[24]^ |
| Graphite-added CrSe_2_ | 3.3910(3) | 5.9144(3) |  | ^[24]^ |

Figure S4. Se K-edge XANES profile of CrSe_2_ and LiCrSe_2_.

Table S4. EXAFS fitting parameters and statistics of Cr K-edge for LiCrSe_2_ and CrSe_2_

AFAC fixed at 0.8

| Sample | Shell | N | r/ Å | σ^2^/ Å | R (fit) | Ref. |
| --- | --- | --- | --- | --- | --- | --- |
| CrSe_2_ | Cr-Se | 6 | 2.47 | 0.0069 | 0.0076 | This work |
| LiCrSe_2_ | Cr-Se | 6 | 2.54 | 0.0053 | 0.0062 | This work |
|  | Cr-Cr | 6 | 3.69 | 0.0101 |  |  |
|  | Cr-Se | 6 | 4.50 | 0.0189 |  |  |
| CrSe_2_ | CrSe_2_ | 6 | 2.4669 |  |  | ^[22]^ |
| LiCrSe_2_ | CrSe_2_ | 6 | 2.5401 |  |  | ^[22]^ |


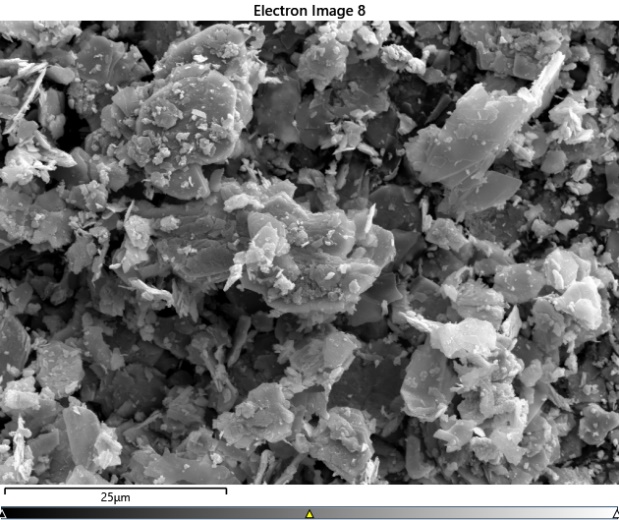

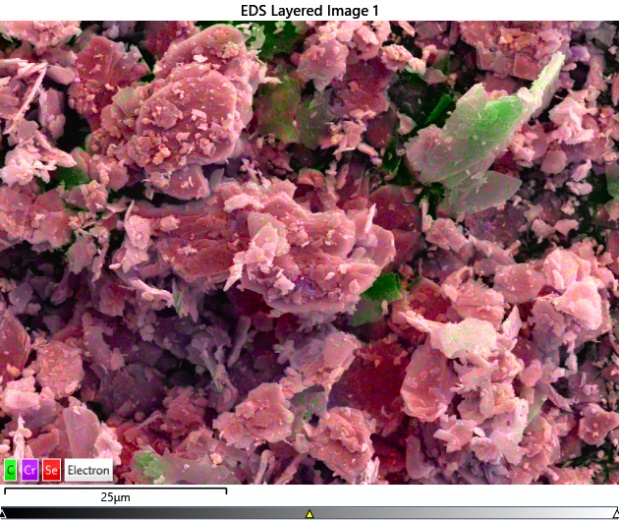

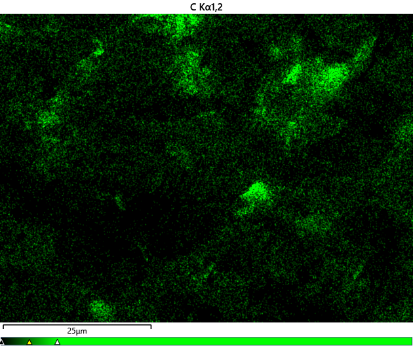

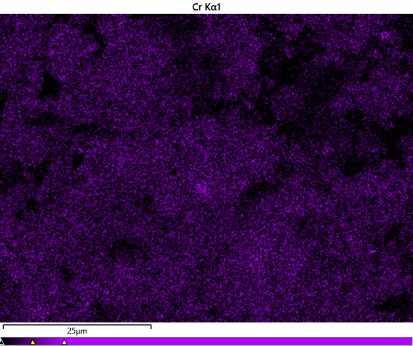

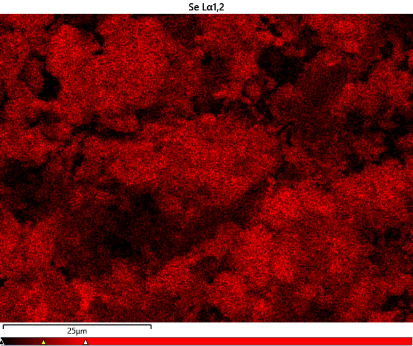


Figure S5 SEM and EDS mapping images of the CrSe_2_ sample obtained via iodine deintercalation from LiCrSe_2_. Individual EDS elemental maps for C (green), Cr (purple), and Se (red).


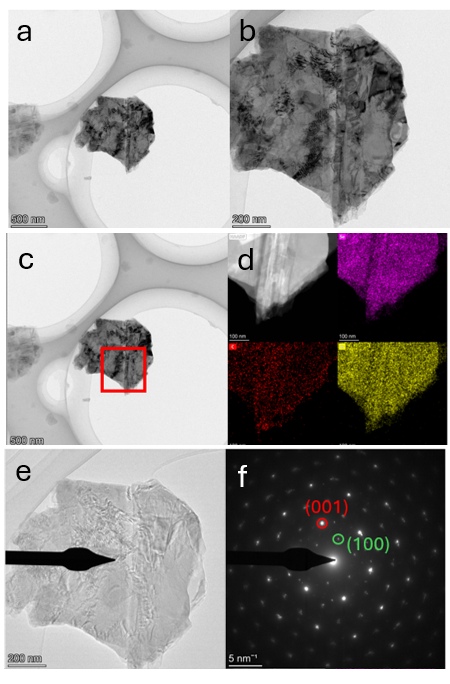


Figure S6. TEM characterization of CrSe_2_. (a, b) Low- and high-magnification TEM images showing the layered morphology of a CrSe_2_ flake. (c) TEM image of the selected region, with (d) corresponding EDX elemental maps showing the distribution of C (red), Cr (yellow), and Se (purple). (e) SAED pattern collected from the flake, indexed to the [001] zone axis of the trigonal CrSe_2_ phase (space group *P*-3*m*1), confirming its single-crystalline nature.


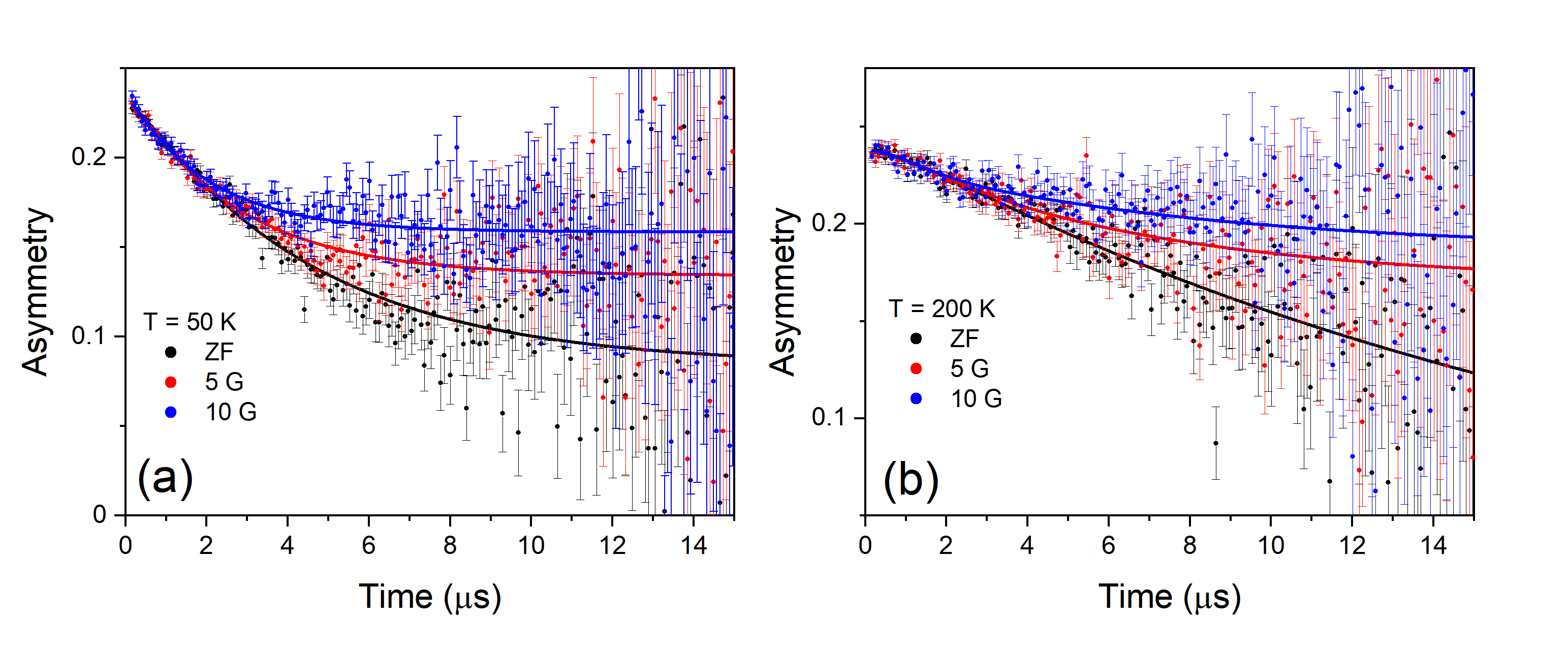


Figure S7. ZF and LF µ^+^SR time spectra at 5 G and 10 G, measured at (a) 50 K and (b) 200 K. The solid lines represent the best fit using formula (3).


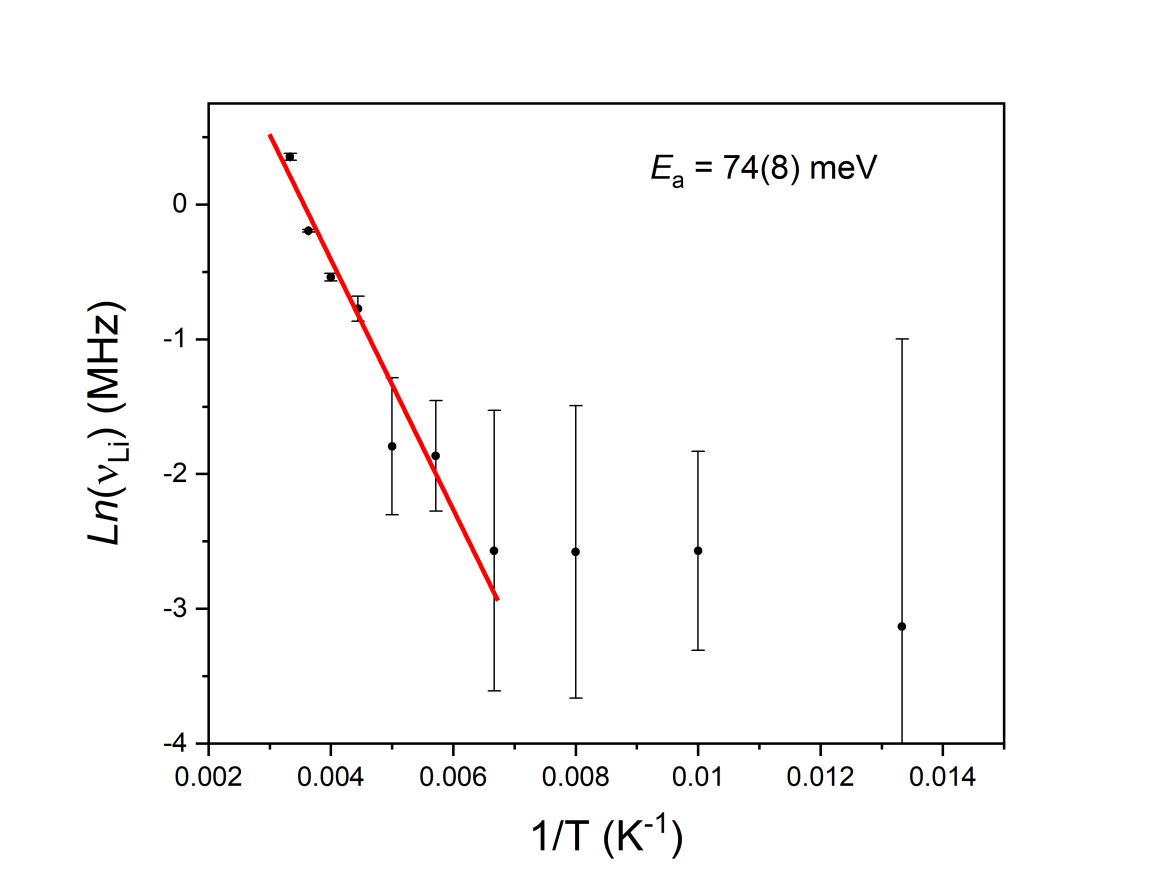


Figure S8. Arrhenius plot of the temperature dependence of the Li-ion hopping rate (ν_Li_), which yield an activation energy of $E_{a} = 74 (8)$ meV.

Figure S9. The GCD profiles for the 2^nd^ cycle of CrSe_2_ recorded at different cut-off voltages (0.75 V; 1 V; 1.25 V, 1.5 V, and 2 V), and the inset photograph shows the reddish discoloration of the separator after the 2^nd^ discharge.

Figure S10. The cycling test of CrSe_2_ at different potential ranges at 0.5C.

Figure S11. The second discharge capacities of CrSe_2_ measured with different lower cut-off voltage

Figure S12. The CV of first three cycles at 0.2 mV s^–1^

Figure S13. The linear relationship between peak current and the square root of scan rate.

Table S5. Li^+^ diffusion coefficients of *P*-3*m*1 TMDs.

| Materials | Li^+^ diffusion coefficient | References |
| --- | --- | --- |
| CrSe_2_ | 7.69 × 10^–9^ cm^2^ s^–1^ (anode)  9.71 × 10^–9^ cm^2^ s^–1^ (cathode) | This work |
| CrSeS | 4.56 × 10^–9^ cm^2^ s^–1^ (anode)  3.24 × 10^–9^ cm^2^ s^–1^ (cathode) | ^[25]^ |
| TiS_2_ | 1.2 × 10^–9^ cm^2^ s^–1^ | ^[26]^ |
| VS_2_ | ~ 1 × 10^–10^ cm^2^ s^–1^ | ^[27]^ |
| TiSe_2_ | ~ 5 × 10^–10^ cm^2^ s^–1^ | ^[28]^ |

Figure S14. Rate performances of CrSe_2_

Figure S15. Selected EIS test points during the first discharge.





Figure S16. The partial density of states of the valence band at various levels of lithiation. For x>0.5 only very few states are available above the fermi level E__F_ indicating reduced electronic conductivity. The band gap fully opens at x=1.0, Egap=1.48 eV.

Figure S17. Temperature-dependent bulk resistivity of LiCrSe_2_ measured using a two-electrode configuration on a pressed pellet inside an Ar-filled glove box. Error bars represent the standard deviation obtained from three independent measurements performed on the same pellet.

Figure S18. *In operando* XRD contour of CrSe_2_ for first two cycles.

Figure S19 Simulated CrSe_2_ XRD pattern based ICSD10313

Figure S20 Simulated LiCrSe_2_ XRD pattern based ICSD10313


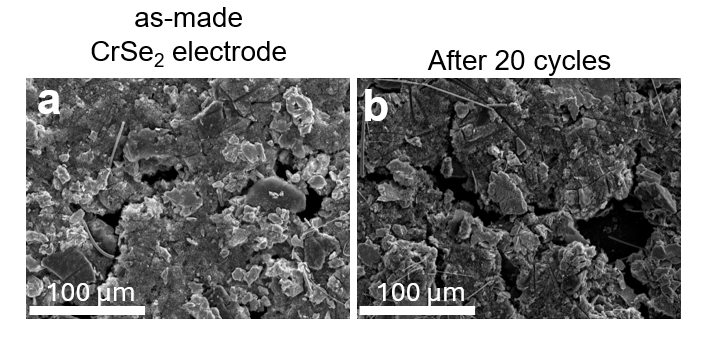


Figure S21. SEM images of CrSe_2_ electrodes (a) in the as-made state and (b) after 20 galvanostatic charge–discharge cycles at 0.5 C. The electrodes were recovered from coin cells disassembled inside an argon-filled glovebox, rinsed with EC/DMC (1:1, v/v) to remove residual electrolyte, and sputter-coated with Au prior to imaging to minimize charging effects. The as-made electrode shows a broad particle-size distribution, which is largely preserved after cycling. After 20 cycles, the electrode exhibits similar particle sizes and overall morphology, with no clear evidence of particle fracture, pulverization, or severe mechanical degradation at the micrometre scale, indicating that the electrode integrity is maintained under the tested cycling conditions. Scale bars: 100 µm.


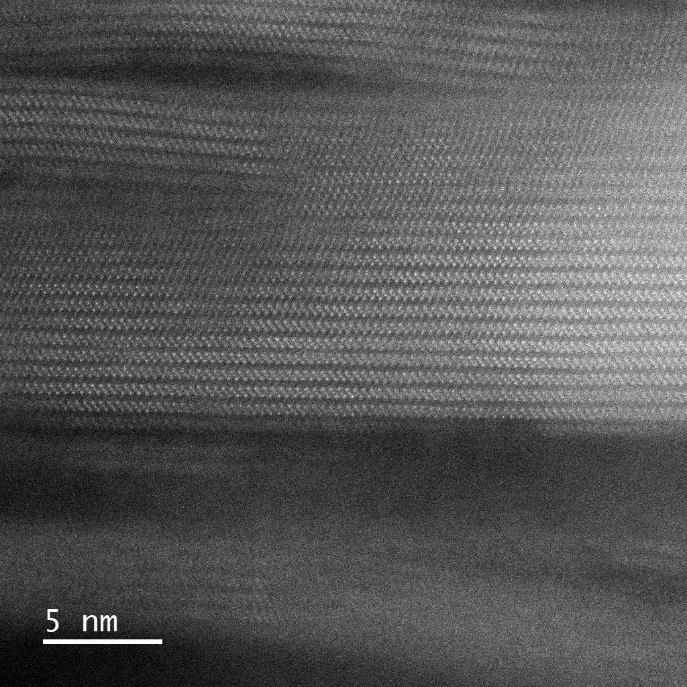


Figure S22. HRTEM image of a CrSe_2_ particle extracted from an electrode at an intermediate lithiation state (x ≈ 0.5 in Li_x_CrSe_2_)

**References**

[1] B. H. Toby, R. B. Von Dreele, *J. Appl. Cryst.* **2013**, *46*, 544-549. https://doi.org/10.1107/S0021889813003531.

[2] B. Ravel, M. Newville, *J. Synchrotron Radiat.* **2005**, *12*, 537-541. https://doi.org/10.1107/S0909049505012719.

[3] S. R. Giblin, S. P. Cottrell, P. J. C. King, S. Tomlinson, S. J. S. Jago, L. J. Randall, M. J. Roberts, J. Norris, S. Howarth, Q. B. Mutamba, N. J. Rhodes, F. A. Akeroyd, *Nucl. Instrum.Methods Phys.Res. A* **2014**, *751*, 70-78. https://doi.org/10.1016/j.nima.2014.03.010.

[4] O. Arnold, J. C. Bilheux, J. M. Borreguero, A. Buts, S. I. Campbell, L. Chapon, M. Doucet, N. Draper, R. F. Leal, M. A. Gigg, V. E. Lynch, A. Markvardsen, D. J. Mikkelson, R. L. Mikkelson, R. Miller, K. Palmen, P. Parker, G. Passos, T. G. Perring, P. F. Peterson, S. Ren, M. A. Reuter, A. T. Savici, J. W. Taylor, R. J. Taylor, R. Tolchenoy, W. Zhou, J. Zikoysky, *Nucl. Instrum.Methods Phys.Res. A* **2014**, *764*, 156-166. https://doi.org/10.1016/j.nima.2014.07.029.

[5] a)N. Matsubara, E. Nocerino, O. K. Forslund, A. Zubayer, K. Papadopoulos, D. Andreica, J. Sugiyama, R. Palm, Z. Guguchia, S. P. Cottrell, T. Kamiyama, T. Saito, A. Kalaboukhov, Y. Sassa, T. Masese, M. Mansson, *Sci Rep* **2020**, *10*, 18305. https://doi.org/10.1038/s41598-020-75251-x; b)A. C. S. Jensen, E. Olsson, H. Au, H. Alptekin, Z. Yang, S. Cottrell, K. Yokoyama, Q. Cai, M.-M. Titirici, A. J. Drew, *J. Mater. Chem. A* **2020**, *8*, 743-749. https://doi.org/10.1039/c9ta10113f.

[6] a)G. Kresse, D. Joubert, *Phys. Rev. B* **1999**, *59*, 1758-1775. https://doi.org/10.1103/PhysRevB.59.1758; b)G. Kresse, J. Furthmuller, *Phys. Rev. B Condens. Matter* **1996**, *54*, 11169-11186. https://doi.org/10.1103/physrevb.54.11169; c)G. Kresse, J. Hafner, *Phys. Rev. B Condens. Matter* **1993**, *47*, 558-561. https://doi.org/10.1103/physrevb.47.558.

[7] P. E. Blochl, *Phys. Rev. B Condens. Matter* **1994**, *50*, 17953-17979. https://doi.org/10.1103/physrevb.50.17953.

[8] J. P. Perdew, K. Burke, M. Ernzerhof, *Phys. Rev. Lett.* **1996**, *77*, 3865-3868. https://doi.org/10.1103/PhysRevLett.77.3865.

[9] J. P. Perdew, A. Ruzsinszky, G. I. Csonka, O. A. Vydrov, G. E. Scuseria, L. A. Constantin, X. Zhou, K. Burke, *Phys. Rev. Lett.* **2008**, *100*, 136406. https://doi.org/10.1103/PhysRevLett.100.136406.

[10] J. Sun, A. Ruzsinszky, J. P. Perdew, *Phys. Rev. Lett.* **2015**, *115*, 036402. https://doi.org/10.1103/PhysRevLett.115.036402.

[11] a)H. W. Peng, Z. H. Yang, J. P. Perdew, J. W. Sun, *Phys. Rev. X* **2016**, *6*. https://doi.org/10.1103/PhysRevX.6.041005; b)R. Sabatini, T. Gorni, S. de Gironcoli, *Phys. Rev. B* **2013**, *87*. https://doi.org/10.1103/PhysRevB.87.041108; c)S. Grimme, J. Antony, S. Ehrlich, H. Krieg, *J. Chem. Phys.* **2010**, *132*, 154104. https://doi.org/10.1063/1.3382344.

[12] S. Kumari, D. K. Pradhan, N. R. Pradhan, P. D. Rack, *Emerg. Mater.* **2021**, *4*, 827-846. https://doi.org/10.1007/s42247-021-00214-5.

[13] a)A. V. Krukau, O. A. Vydrov, A. F. Izmaylov, G. E. Scuseria, *J. Chem. Phys.* **2006**, *125*, 224106. https://doi.org/10.1063/1.2404663; b)J. Heyd, G. E. Scuseria, M. Ernzerhof, *J. Chem. Phys.* **2003**, *118*, 8207-8215. https://doi.org/10.1063/1.1564060.

[14] W. Li, J. Doehn, J. Y. Chen, M. Dillenz, M. Sotoudeh, D. M. Pickup, S. R. Luo, R. Parmenter, J. Arbiol, M. Alfredsson, A. V. Chadwick, A. Gross, M. Zarrabeitia, A. Y. Ganin, *J. Mater. Chem. A* **2024**, *12*, 31276-31283. https://doi.org/10.1039/d4ta05114a.

[15] C. J. Bartel, *J. Mater. Sci* **2022**, *57*, 10475-10498. https://doi.org/10.1007/s10853-022-06915-4.

[16] S. Panja, Y. D. Miao, J. Döhn, J. Choi, S. Fleischmann, S. G. Chandrappa, T. Diemant, A. Gross, G. Karkera, M. Fichtner, *Adv. Funct. Mater.* **2025**, *35*. https://doi.org/10.1002/adfm.202413489.

[17] A. Urban, D.-H. Seo, G. Ceder, *npj Comput. Mater.* **2016**, *2*. https://doi.org/10.1038/npjcompumats.2016.2.

[18] D. Sheppard, R. Terrell, G. Henkelman, *J. Chem. Phys.* **2008**, *128*, 134106. https://doi.org/10.1063/1.2841941.

[19] G. Henkelman, B. P. Uberuaga, H. Jónsson, *J. Chem. Phys.* **2000**, *113*, 9901-9904. https://doi.org/10.1063/1.1329672.

[20] E. Nocerino, S. Kobayashi, C. Witteveen, O. K. Forslund, N. Matsubara, C. Tang, T. Matsukawa, A. Hoshikawa, A. Koda, K. Yoshimura, I. Umegaki, Y. Sassa, F. O. von Rohr, V. Pomjakushin, J. H. Brewer, J. Sugiyama, M. Månsson, *Commun. Mater.* **2023**, *4*. https://doi.org/10.1038/s43246-023-00407-x.

[21] W. P. Xu, S. Ali, Y. J. Jin, X. Z. Wu, H. Xu, *ACS Appl. Electron. Mater.* **2020**, *2*, 3853-3858. https://doi.org/10.1021/acsaelm.0c00686.

[22] S. Kobayashi, N. Katayama, T. Manjo, H. Ueda, C. Michioka, J. Sugiyama, Y. Sassa, O. K. Forslund, M. Mansson, K. Yoshimura, H. Sawa, *Inorg. Chem.* **2019**, *58*, 14304-14315. https://doi.org/10.1021/acs.inorgchem.9b00186.

[23] W. Li, J. Döhn, J. Chen, M. Dillenz, M. Sotoudeh, D. M. Pickup, S. Luo, R. Parmenter, J. Arbiol, M. Alfredsson, A. V. Chadwick, A. Groß, M. Zarrabeitia, A. Y. Ganin, *J. Mater. Chem. A* **2024**, *12*, 31276-31283. https://doi.org/10.1039/d4ta05114a.

[24] W. Li, N. Wolff, A. K. Samuel, Y. S. Wang, V. P. Georgiev, L. Kienle, A. Y. Ganin, *Chemelectrochem* **2023**, *10*. https://doi.org/10.1002/celc.202300428.

[25] S. Y. Yang, D. R. Shi, T. Wang, X. Y. Yue, L. Zheng, Q. H. Zhang, L. Gu, X. Q. Yang, Z. Shadike, H. Li, Z. W. Fu, *J. Mater. Chem. A* **2020**, *8*, 25739-25745. https://doi.org/10.1039/d0ta08012h.

[26] S. Fleischmann, H. Shao, P. L. Taberna, P. Rozier, P. Simon, *ACS Energy Lett.* **2021**, *6*, 4173-4178. https://doi.org/10.1021/acsenergylett.1c01934.

[27] D. Q. Xin, S. D. He, X. D. Zhang, R. S. Li, W. Y. Qiang, S. J. Duan, Q. Lou, K. L. Deng, Z. F. Cheng, M. G. Xia, *J. Energy Storage.* **2023**, *72*. https://doi.org/10.1016/j.est.2023.108688.

[28] P. Li, X. B. Zheng, H. X. Yu, G. Q. Zhao, J. Shu, X. Xu, W. P. Sun, S. X. Dou, *Energy Storage Mater.* **2019**, *16*, 512-518. https://doi.org/10.1016/j.ensm.2018.09.014.
